# Emergent Antiferroelectric Ordering and the Coupling of Liquid Crystalline and Polar Order

**DOI:** 10.1002/smsc.202400189

**Published:** 2024-07-03

**Authors:** Jordan Hobbs, Calum J. Gibb, Richard J. Mandle

**Affiliations:** ^1^ School of Physics and Astronomy University of Leeds Leeds LS2 9JT UK; ^2^ School of Chemistry University of Leeds Leeds LS2 9JT UK

**Keywords:** ferroelectrics, liquid crystals, soft matter, polar order

## Abstract

Polar liquid crystals possess 3D orientational order coupled with unidirectional electric polarity, yielding fluid ferroelectrics. Such polar phases are generated by rod‐like molecules with large electric dipole moments. 2,5‐Disubstituted 1,3‐dioxane is commonly employed as a polar motif in said systems, and herein it is shown to suffer from thermal instability as a consequence of equatorial‐trans to axial‐trans isomerism at elevated temperatures. Isosteric building blocks are utilized as potential replacements for the 1,3‐dioxane unit, and in doing so new examples of fluid ferroelectric systems are obtained. For binary mixtures of certain composition, the emergence of a new fluid antiferroelectric phase, a finding not observed for either of the parent molecules, is observed. This study also reveals a critical tipping point for the emergence of polar order in otherwise apolar systems. These results hint at the possibility for uncovering new highly ordered polar liquid‐crystalline phases and delineate distinct transition mechanisms in orientational and polar ordering.

## Graphical abstract

Replacing the problematic 2,5‐disubstituted‐1,3‐dioxane unit of DIO with a range of isosteric building blocks affords a family of materials which show a fluid antiferroelectric state in the binary phase diagrams, despite such a phase not being observed for any of the pure materials.

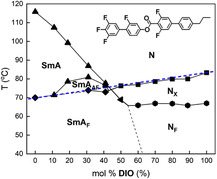

## 1 Introduction

**Figure 1:**
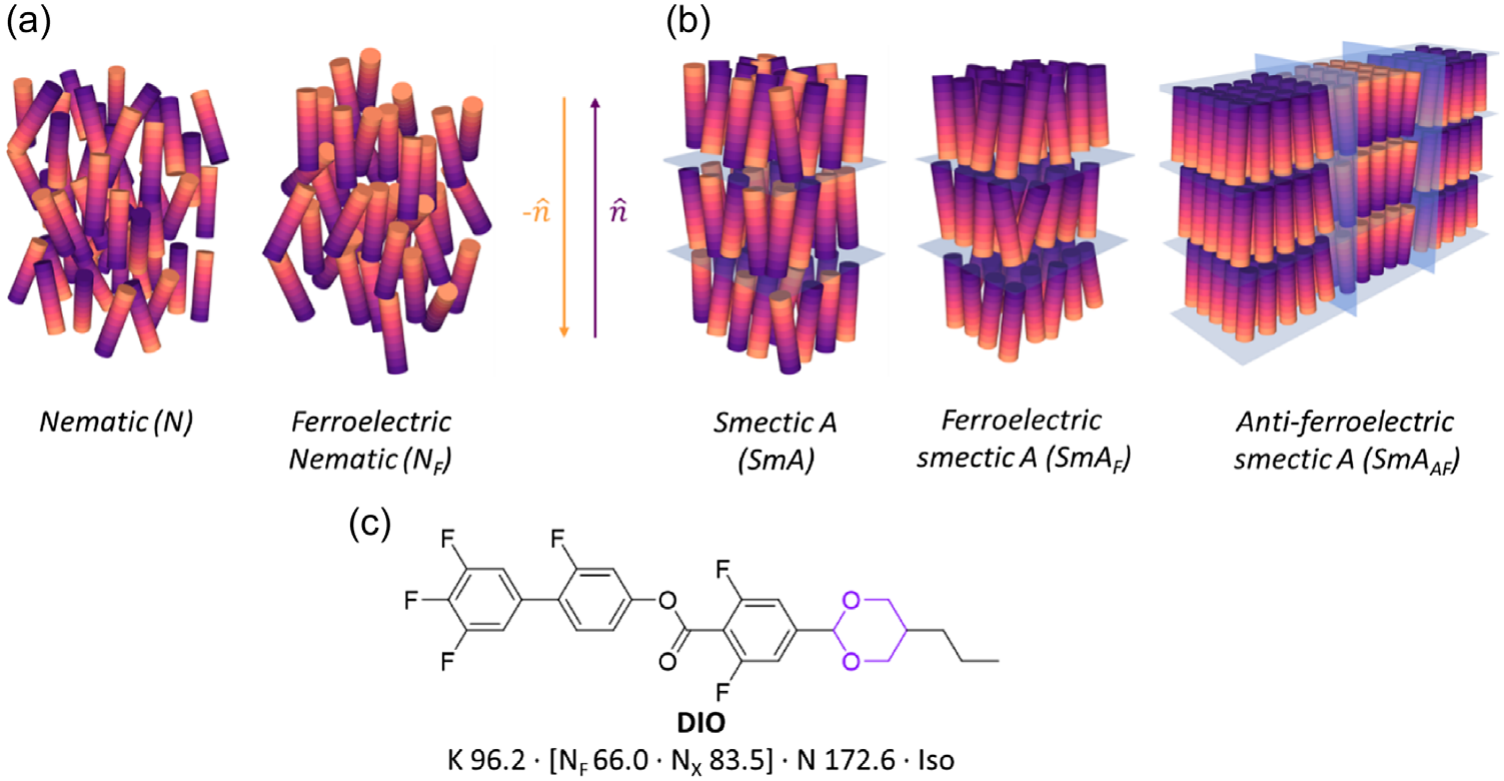
a) Schematic representations of the nematic (N) [left] and ferroelectric nematic (*N*_F_) [right] phases. b) Schematic representations of the SmA phase [left] and, an example of a lamellar phase with polar order, the SmA_F_ phase, and c) the chemical structure of the archetypal *N*_F_ material DIO.^[^ ^4^ ^]^ The 1,3‐dioxane unit is highlighted in purple.

The nematic (N) liquid‐crystalline (LC) phase consists of molecules or particles which lack translational order but retain a degree of orientational ordering, with the molecules aligning along a unit vector termed the director ($\hat{n}$) (**Figure** **1**a). The conventional N phase possesses inversion symmetry (i.e., the nematic director ($\hat{n}$) is equivalent to its reciprocal, $\hat{n}=-\hat{n}$) and so is apolar, even when formed from highly polar molecules. While subject of some interest at the turn of the 20th century,^[^ ^1^ ^]^ an inherently polar N phase lacking inversion symmetry (i.e., $\hat{n}\neq -\hat{n}$) (Figure 1a) was only experimentally discovered in 2017 simultaneously and independently in two different materials: **RM734** ^[^ ^2^, ^3^ ^]^ and **DIO**.^[^ ^4^ ^]^ Now referred to as the ferroelectric nematic (*N*_F_) phase, the emergence of a new nematic phase at equilibrium has garnered significant excitement among the scientific community^[^ ^5^, ^6^, ^7^, ^8^, ^9^, ^10^, ^11^, ^12^ ^]^ and has been promised to “remake nematic science and technology”.^[^ ^13^ ^]^ Given the low energy cost of elastic deformation of many LC, polar variants of other “transitional” LC phases have now been observed including ferroelectric^[^ ^14^, ^15^, ^16^, ^17^ ^]^ and antiferroelectric^[^ ^16^ ^]^ analogues of the smectic A (SmA) phase, denoted as the SmA_F_ and SmA_AF_ phases, respectively (Figure 1b), as well as more recently spontaneously chiral and tilted nematic and smectic phases.^[^ ^16^, ^18^ ^]^

LCs exhibiting polar behavior can be considered as being constructed from multiple polar fragments, such as the 2,5‐disubstituted 1,3‐dioxane found in DIO (Figure 1c). The 2,5‐disubstituted dioxane makes a modest contribution to the molecular electric dipole moment (≈1 D at MP2/6‐311 + G(d,p)) and is found in materials exhibiting the *N*_F_, *N*_X_ (sometimes labeled as SmZ_A_ or *N*_S_),^[^ ^4^ ^]^ *N*_TBF_,^[^ ^18^ ^]^ SmA_F_,^[^ ^14^ ^]^ and the ${\text{SmC}}_{P}^{H}$ phase.^[^ ^16^ ^]^ However, 2‐aryl‐1,3‐dioxane containing LCs (such as **DIO**) is widely known to have relatively poor thermal stability yet this is seldom explained beyond noting some structural rearrangement may occur above some temperature.

Herein, we present a detailed analysis of the structural rearrangement that does indeed occur for 2‐aryl‐1,3‐dioxane containing LCs and explore some alternatives to the 1,3‐dioxane unit of **DIO**. We generally observe a destabilization of polar phases except for a single compound which exhibits a SmA_F_ phase. Mixtures of this compound with DIO are fabricated which show an emergent SmA_AF_ phase. By studying various binary mixtures exhibiting polar LC behavior, we also shed light onto the relative coupling between LC and polar order for mesogenic materials.

## 2 Results and Discussion

**Figure 2:**
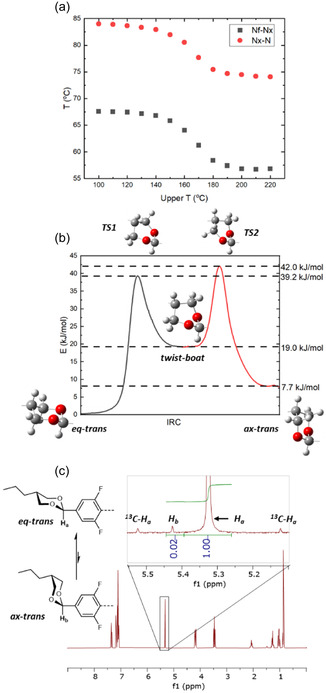
a) Transition temperature of **DIO** which has been heated to the temperature marked as “upper T” as determined via DSC. b) IRC trace of **DIO** showing the eq‐trans to ax‐trans isomerisation via half‐chair (TS) and twist boat forms with only the dioxane fragment shown here. c) NMR spectra of **DIO** which was heated to 190 °C showing the emergence of a second peak associated with the ax‐trans form of **DIO**; relevant integrated peak areas are given under each peak.

It is known that **DIO “**degrades” when heated to temperatures above 120 °C, and this has been suggested to be a *trans*‐cis structural isomerism concerning the 1,3‐dioxane unit.^[^ ^4^, ^19^, ^20^ ^]^ A sample of **DIO** was subjected to repeated heat/cool via DSC cycles, varying the maximum temperature (100–220, 10 °C increments, Figure S1, Supporting Information). Repeated heating and cooling cycles show no decrease in the onset of the ferroelectric to antiferroelectric and antiferroelectric to nematic phase transitions (*T*_NF‐NX_ and *T*_NX‐N_, respectively) until 140 °C, where subsequent cycles result in decreased mesophase transition temperatures (**Figure** **2**a). The decrease is continuous until 190 °C where the transition temperatures plateaus at ≈10 °C below their initial values; the plateau implies an equilibrium is attained as opposed to continuous thermal decomposition. The proposed *cis*/*trans* isomerization of 1,3‐dioxane cannot occur without breaking and remaking C—O bonds; thus, we consider this unlikely at the temperatures in question. All 2,5‐di‐substituted 1,3‐dioxanes have two principal *trans* conformations, *trans* equatorial (*eq‐trans*) and *trans* axial (*ax‐trans*), which can interconvert via twist‐boat states and half‐chair transition state conformations (Figure S2, Supporting Information).^[^ ^21^ ^]^ Calculation of the intrinsic reaction coordinate pathway (IRC, at the B3LYP‐GD3BJ/cc‐pVTZ level of density functional theory (DFT)) confirms the *eq‐trans* to *ax‐trans* assignment and supports the isomerization proceeding by this mechanism (Figure 2b). The NMR spectrum of a sample of **DIO** heated above 190 °C showed shifts in resonances consistent with partial isomerization from the *eq‐trans* form to the *ax‐trans* form (Figure 2c). A comparison of the peak areas of the two singlets reveals that the *ax‐trans* conformation comprises only 3% of the resultant mixture after heating, consistent with the modest decrease in the values of *T*_NF‐NX_ and *T*_NX‐N_ observed by DSC as well as the 7.7 kJ mol^−1^ energy difference as calculated from the DFT IRC trace.

**Figure 3:**
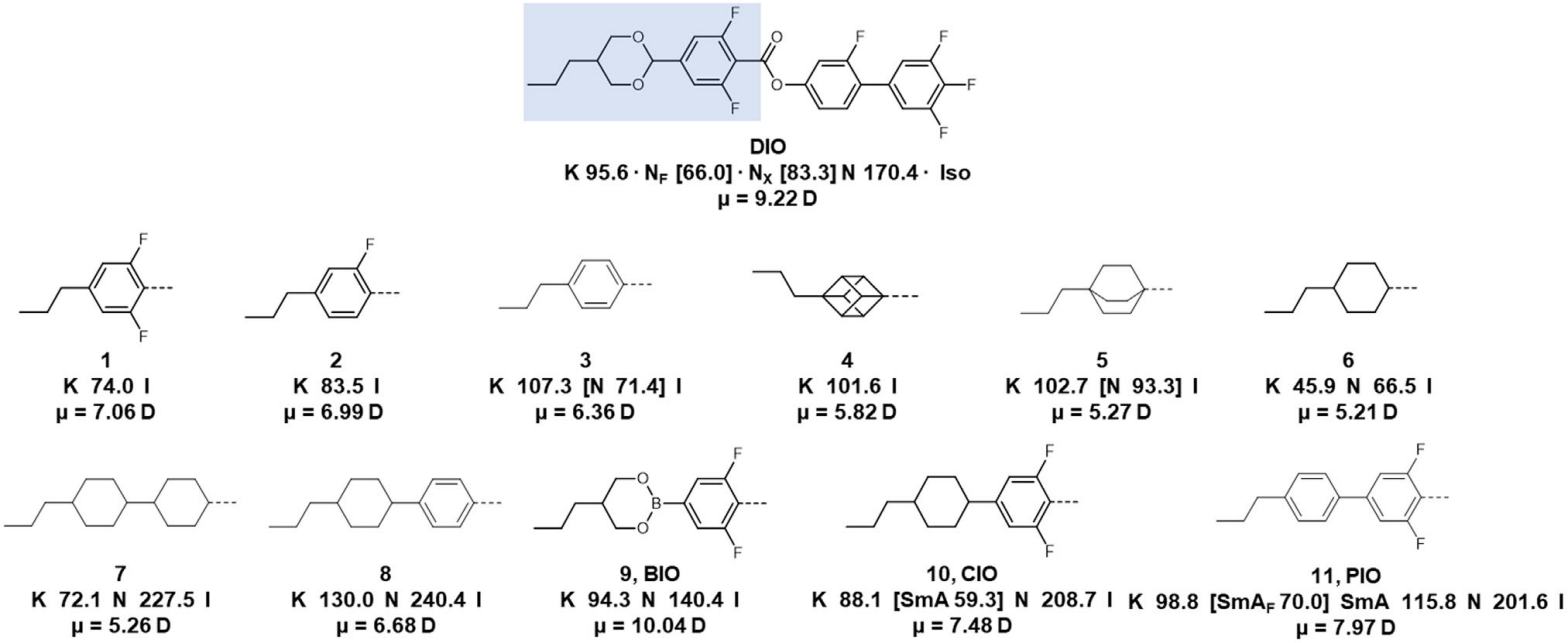
Chemical structures of the terminal groups chosen as alternatives to the 1,3 dioxane ring seen in **DIO** for this work, their associated transitional properties, and longitudinal molecular dipole moments (μ, at the B3LYP‐GD3BJ/cc‐pVTZ level of DFT) of compounds **1**‐**11**. The transition temperatures given are quoted in °C with ‘[ ]’ indicating a monotropic phase transition. Specific compounds of interest are given short generic names (**9**: BIO, **10**: CIO, **11**: PIO).

With the thermal instability of the 1,3‐dioxane unit now understood, we move to study alternative ring systems with a view to enhancing the thermal stability of the parent molecule, **DIO**. Given the number of possible end‐groups we could have chosen, we imposed two constraints to focus our investigation. The first is that all end‐groups possess a propyl terminal chain as this has been shown to be an appropriate length to facilitate favorable head‐to‐tail correlations between neighboring molecules^[^ ^2^, ^22^, ^23^ ^]^ The second is that all end‐groups must contribute to the overall longitudinal dipole moment of the molecule (μ) as a large value of μ is thought necessary for the formation of polar mesophases.^[^ ^9^, ^24^ ^]^ The chemical structures, their associated transitional data and their molecular dipole moment are shown in **Figure** **3**. Transitional data and obtained DFT parameters are given in tabulated form in the ESI (Table S1 and S2, Supporting Information, respectively). A detailed description of the chemical synthesis of these materials is provided in the ESI to this article.

We synthesized six materials whereby the 2‐(3,5‐difluorophenyl)‐1,3‐dioxane unit is supplanted by a “single ring” bearing propyl terminal chain (**1–6**). These compounds variously incorporate aromatic (**1**–**3**) and/or fluorinated (**1**, **2**), saturated (**4**–**6**), and/or bi/polycyclic (**4**, **5**) ring systems. Compound **1** can be considered as DIO minus the 1,3‐dioxane ring; however, **1** is nonmesogenic as is the closely related compound **2**. The closely related compound **3**, differing from **1** and **2** in terms of the number of fluorine atoms on the benzoate unit, exhibits a monotropic nematic phase. Replacement of the 1,4‐benzene ring of **3** with other isosteric groups gave mixed results: the 1,4‐cubane (**4**) is nonmesogenic; the 1,4‐bicyclo[2.2.2]octane (**5**) and the *trans* 1,4‐cyclohexane (**6**) are both nematic. Compounds **3**, **5,** and **6** exhibit nematic phases with significantly reduced clearing points relative to the parent **DIO**, and we conjecture that this is related to the decreased molecular length.

We therefore prepared materials with similar aspect ratios to **DIO** by incorporating an additional ring unit. Compound **7** is related to **6** but bears an additional *trans* 1,4‐cyclohexane unit and exhibits a considerable increase in *T*_N‐I_ (over 160 °C) with only a modest increase in melting point. Similarly, compound **8** is analogous to **3** but with the addition of a *trans* 1,4‐cyclohexane (or alternatively, analogous to **6** but with an additional 1,4‐benzene); dramatic increases in clearing point are observed, as well as significantly higher melting point. For both **7** and **8,** the molecular electric dipole moments are comparable with the parent three‐ring systems (**3** or **6**) and the mesophases observed are nonpolar. Next, we sought to increase the polarity of the materials (and the molecular electric dipole moment) by use of heterocycles and/or fluorination. Compound **9** (aka **BIO**) bears a 1,3,2‐dioxaborinane ring as well as a 3,5‐difluorobenzene; this combination yields a larger molecular dipole moment than **DIO**. However, **BIO** is both unstable with respect to air and water and only exhibits solely conventional nematic behavior.

**Figure 4:**
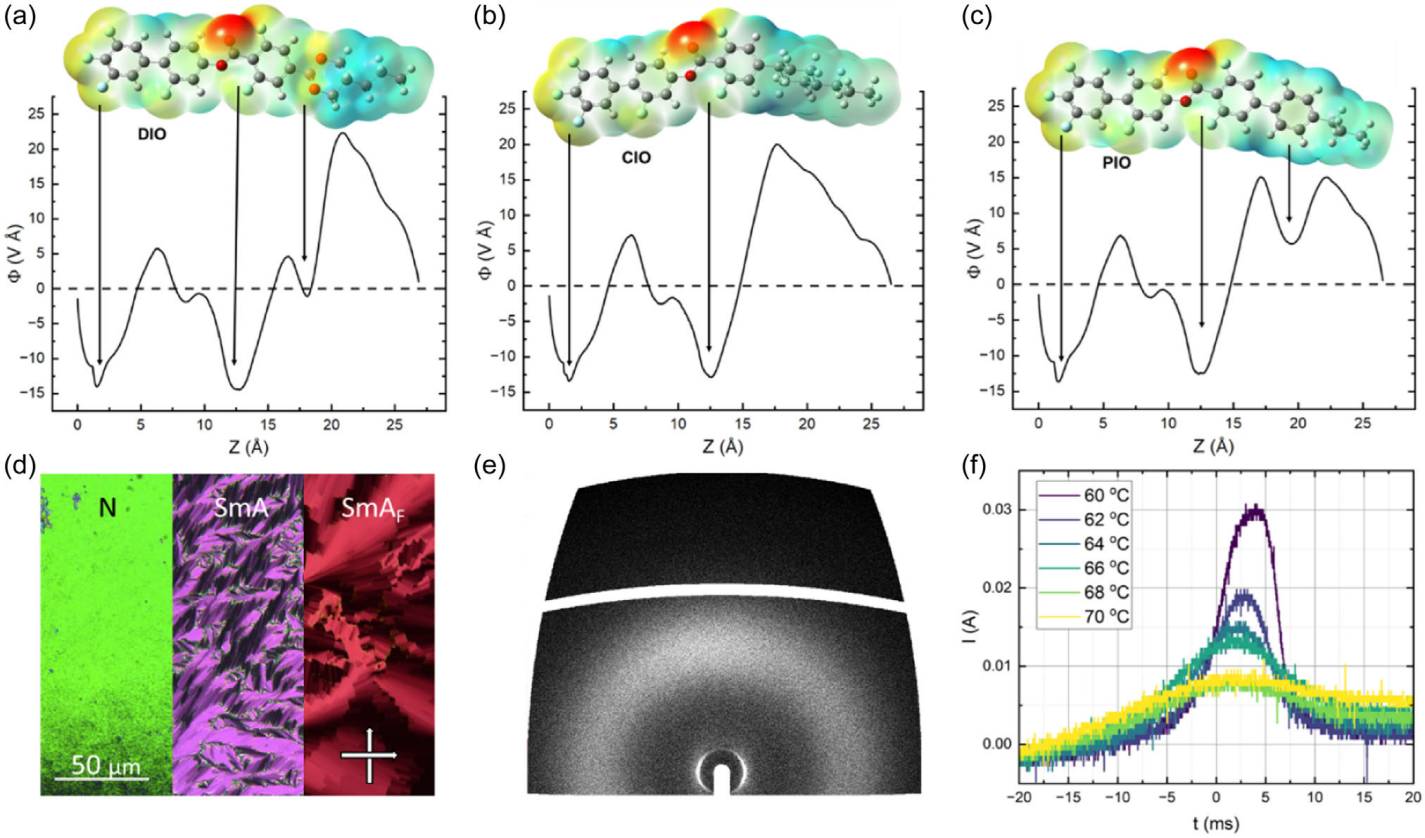
Electrostatic potential, scaled by contour length (discussed in ESI, Supporting Information), along the *z*‐axis of the molecule at an electron density isosurface of 0.0004 calculated at the B3LYP‐GD3BJ/cc‐pVTZ level of DFT for a) **DIO**, b) **CIO**, and c) **PIO**. d) POM micrographs depicting the phase sequence of **PIO**. Images were taken within 8 μm cells with no alignment layer, e) 2‐D X‐ray scattering pattern of **PIO** in the SmA phase and f) current response of **PIO**. Given the propensity for crystallizations of the sample, rapid cooling of the sample (>100 °C) is necessary to study the phase. The single polarization peak indicates the ferroelectric nature of **PIO** at ≈70 °C although the magnitude of the peak is unreliable due to partial sample crystallizations.

The 1,3‐dioxane of DIO is replaced with cyclohexane in compound **10** (aka **CIO**). While this leads to a somewhat lower molecular electric dipole moment (7.48 D vs. 9.22 D for **DIO**, both at the B3LYP‐GD3BJ/cc‐pVTZ level of DFT), **CIO** has a significantly higher clearing point (and a slightly lower melting point) than **DIO**. We find that **CIO** exhibits a monotropic conventional SmA phase below an enantiotropic N phase which was characterized by the observation of focal conic fans (Figure S4, Supporting Information) by POM. We again, however, see no evidence of any polar mesophase behavior for **CIO** in either POM observations or Ps measurements despite possessing a similar molecular shape and a comparable molecular dipole moment. Work by Madhusudana has previously suggested that a longitudinal surface charge density wave along the length of the molecule is important in stabilizing parallel molecular orientations.^[^ ^25^ ^]^ The limited anisotropic polarizability of the cyclohexane moiety in **CIO** may therefore be detrimental to the formation of polar mesophases. The electrostatic potential energy surface was calculated using B3LYP‐GD3BJ/cc‐pVTZ level of DFT. By radially averaging and plotting the electrostatic potential as a function of the *z*‐axis (described in more detail in the Experimental Section), we can see that electronically speaking, the cyclohexane ring acts essentially as an extension of the terminal chain reducing the prominence of the longitudinal surface charge density wave (**Figure** **4**a,b). Replacing the cyclohexane moiety with a phenyl ring (compound **11** aka **PIO**) introduces additional anisotropic polarizability, returning somewhat of the oscillatory charge density wave found for **DIO** (Figure 4c and S5, Supporting Information) while the increased conjugation leads to a somewhat higher molecular electric dipole moment, and so we considered this to be a promising candidate for exhibiting polar LC phases. We find **PIO** to have a slightly higher *T*_NI_ and *T*_m_ than **DIO**. Significant stabilization of smectic ordering is found in **PIO**, with a transition from the N to a SmA phase at 115.8 °C, an increase of around 55 °C compared **CIO**. The assignment of the SmA phase was confirmed by POM via the observation of a focal conic fan texture (Figure 4d) and via X‐ray scattering where the 2D pattern showed diffuse liquid‐like order in the wide angle X‐ray scattering (WAXS) region and intense Bragg scattering in the small angle X‐ray scattering (SAXS) region indicative of a lamella structure (Figure 4e) with a d‐spacing of 2.40 nm corresponding to a monolayer SmA phase. Typically speaking, in apolar liquid lamellar crystals such as 8CB, the replacement of a 1,4‐disubstituted benzene ring with a 2,5‐disubstituted 1,3‐dioxane suppresses the formation of smectic layers, giving only a nematic phase^[^ ^26^ ^]^ and the same behavior would appear to be at play here.

Given its similarity to **DIO**, the lack of polar phase behavior in **PIO** was surprising. DSC measurements and POM cooling at rate of around 10 °C min^−1^ resulted in crystallization around 80 °C, giving only a small window of supercooling to observe any further phase transitions. We hypothesized that any transitions to a polar phase would be at least below 80 °C (the transition to the *N*_X_ phase in **DIO**). We therefore studied **PIO** by POM and measured its response to spontaneous polarization (*P*_S_) while rapidly cooling the sample (>100 °C min^−1^). At ≈70 °C, a transition to polar phase was observed which we assigned to be a SmA_F_ phase by the appearance of a blocky mosaic texture (Figure 4d) and a single peak in the current response indicative of ferroelectric ordering (Figure 4f). These observations are consistent with previous observations of the SmA_F_.^[^ ^14^, ^15^, ^16^ ^]^ Ideally, this phase assignment would be verified via X‐ray scattering and detailed *P*_S_ measurements; however, the stringent cooling requirements prohibited further analysis and so it was envisioned that the formulation of binary mixtures with **DIO** would suppress the melting points sufficiently, allowing for this phase assignment to be verified.

**Figure 5:**
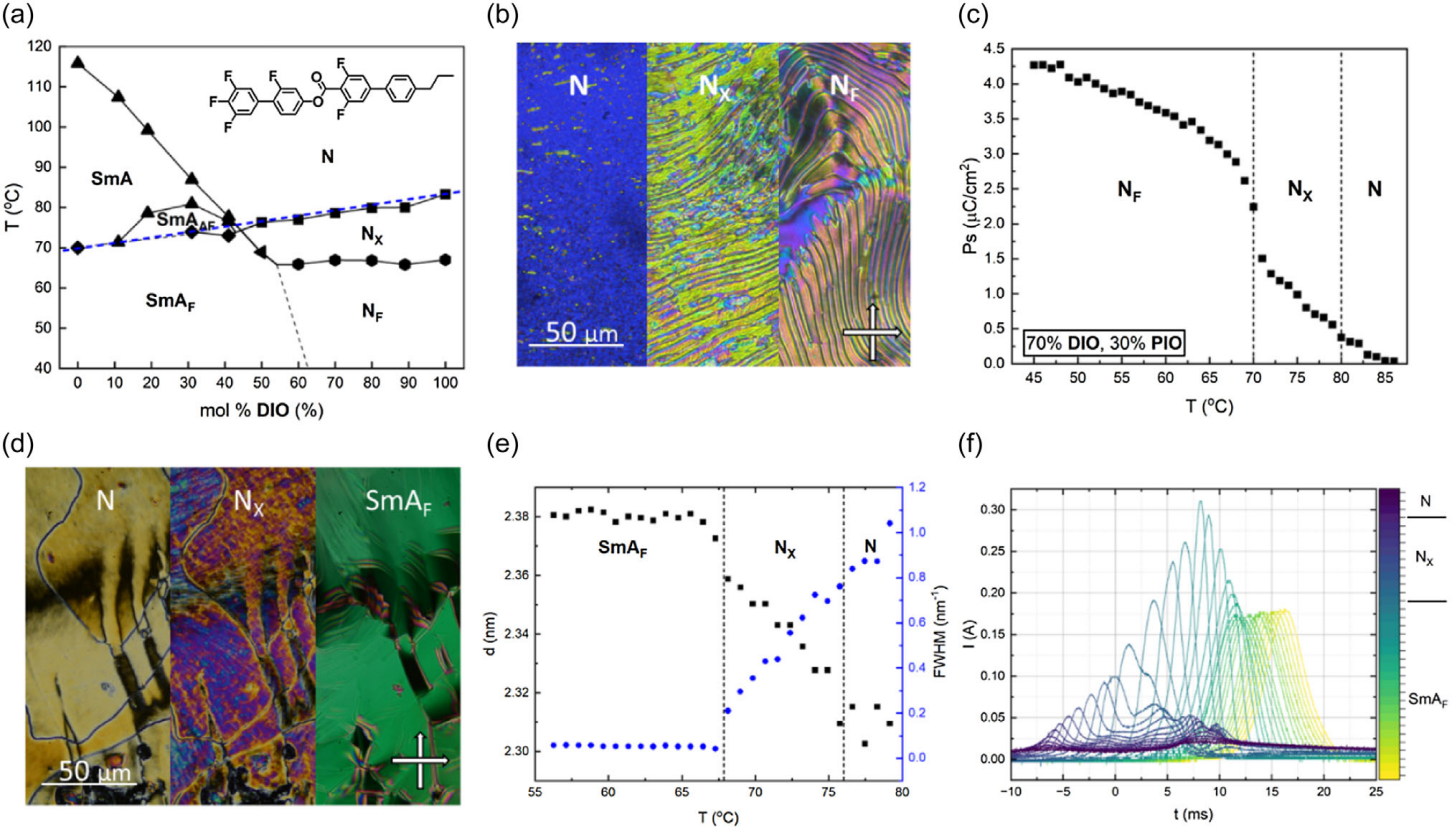
a) Phase diagram for mixtures of **PIO** with **DIO**. b) POM micrographs depicting the phase sequence the mixture comprising 70% **DIO** and 30% **PIO**. Images were taken within 8 μm cells with no alignment layer. c) Variation of *P*_S_ as a function of temperature measured for the mixture comprising 70% **DIO** and 30% **PIO**. d) POM micrographs depicting the blocky texture of the SmA_F_ phase observed for a mixture comprising 50% **DIO** and 50% **PIO**. Images were taken within 8 μm cells with no alignment layer. e) Position and FWHM of the X‐ray scattering peak associated with long molecular axis correlations for a mixture of 50% **DIO** and 50% **PIO**. f) Spontaneous polarization current response of mixtures containing 50% **DIO** and 50% **PIO**.

Mixing **PIO** and **DIO** (**Figure** **5**a) results in a modest reduction in *T*_m_ (≈20 °C reduction from **DIO**), leading to several significant observations. In high concentrations of **DIO,** phase behavior matching that of the dominant molecule is observed which, when combined with the reduction in the values of *T*_m_, leads to mixtures showing enantiotropic *N*_F_ phases which could be supercooled to room temperature in as little as 10 mol% of **PIO**. The *N*_F_ phases were characterized by the observation of banded textures using POM (Figure 5b), characteristic of the *N*_F_ phase, with the assignment further affirmed by the measurement of spontaneous polarization (Figure 5c) which demonstrated a single peak in the current response demonstrative of the *N*_F_ phase. For mixtures comprising 30–60 mol% of **DIO** in **PIO**, a phase with a similar blocky fan POM texture (Figure 5d) is observed, similar to that seen for pure **PIO**. WAXS and SAXS measurements confirm liquid‐like order with a lamellar structure, with the layer normal parallel to the director (Figure 5e). For a 50:50 mixture Bragg scattering was measured at ≈2.30 nm, indicating a slightly reduced layer spacing compared to pure **PIO** (2.40 nm). *P*_S_ measurements confirm that the phase is ferroelectric from the single peak in the current response (Figure 5f). Extrapolation of these transitions shows a linear dependence with the SmA_F_ phase transition assigned for pure **PIO**.

**Figure 6:**
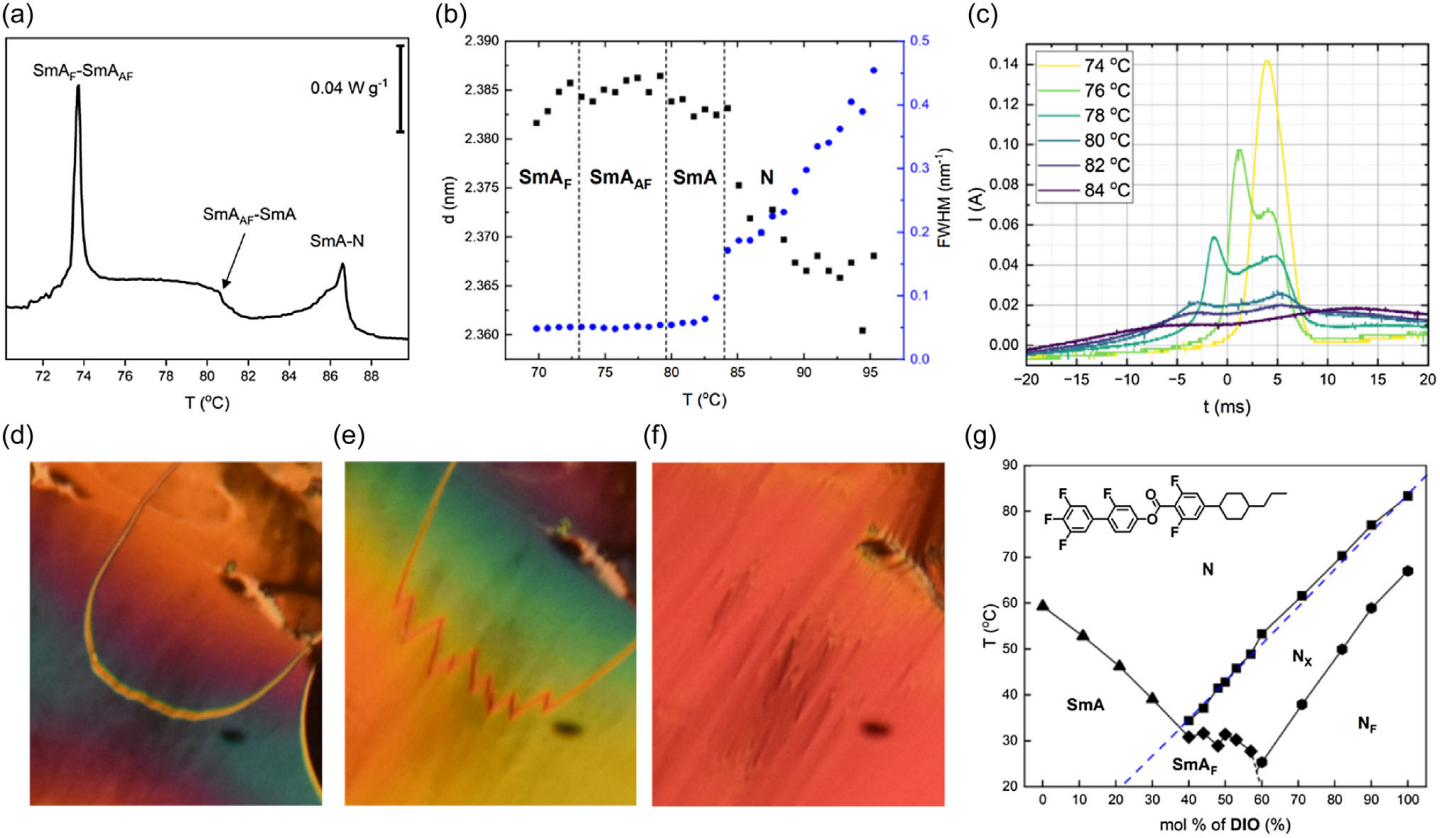
a) DSC thermogram for 30% **DIO** and 70% **PIO** showing the thermal transition into the SmA_AF_ phase. b) Position and FWHM of the X‐ray scattering peak associated with long molecular axis correlations for 30% **DIO** and 70% **PIO**. c) Current response for the SmA_AF_ phase showing the two peaks. d–f) POM micrographs of a mixture of 40% **DIO** and 60% **PIO** showing how a chevron defect evolves between the narrow ≈2 °C window where the sample transitions from N‐SmA‐SmA_AF_. g) Phase diagram between **DIO** and **CIO** with the transition from apolar–polar order marked in blue.

Mixtures fabricated from **PIO** and **DIO** allow for significant supercooling of the SmA_F_ phase below their melting points (circa >15 °C below *T*_m_ by POM). For mixtures comprised between 10 and 40 mol% of **DIO**, conventional N and SmA phases are observed, with a phase transition to a new smectic phase not observed for either **PIO** or **DIO**. Texturally the phase presents with a similar texture to the SmA phase by POM (as described previously). However, a small step‐like transition with a small enthalpy change is observed in the DSC thermograms (**Figure** **6**a). SAXS experiments indicate that the phase has a similar lamellar structure to the preceding SmA phase, with only a very slight change in the layer spacing (2.383 nm in the SmA to 2.385 nm in the 2nd smectic phase) observed at the phase transition (Figure 6b). Measurements of the current response show a transition from apolar ordering in the SmA phase to a double peak upon cooling into the lower‐temperature phase, indicating possible antiferroelectric ordering within the lamellar structure (Figure 6c). Further cooling this phase sees the combining of the double peak into a single peak (ferroelectric‐like ordering) with the blocky focal conic fan texture of the SmA_F_ phase observed by POM. A longitudinal antiferroelectric SmA (SmA_AF_) phase has been demonstrated previously in highly polar LCs^[^ ^16^ ^]^ though the emergence of antiferroelectric ordering here is certainly unexpected. No evidence for phase separations can be seen in either POM or SAXS measurements. Ferroelectric order can be induced in N materials above the transition to polar order^[^ ^26^ ^]^ using electric fields which could explain the antiferroelectric response (peak on increasing voltage associated with induced order while the one on reducing voltage associated with the return to apolar order). However, the distinct thermal transition from the DSC suggests that this is also not the case. At a concentration of 40% **DIO**, the transitions from N to SmA to SmA_AF_ all occur in a very small temperature window and as such additional pretransitional antiferroelectric ordering can be seen in the N phase (as it transitions to the SmA and subsequent SmA_AF_ phase) in the form of chevron defects (Figure 6d–f). This may suggest the formation of a very narrow N_X_ phase^[^ ^27^ ^]^ which may mean the phase order is actually N–*N*_X_‐SmA_AF_ and confirms that the two peaks in the current response for this region are not an electric field‐induced phase transition to ferroelectric ordering and are instead representative of the ground state. It also suggests that the appearance of antiferroelectric order is unrelated to the lamellar order of the SmA phase. While we are not certain as to the molecular origins of SmA_AF_ phase in this case, we suggest that **DIO** and **PIO** possibly have some molecular interaction which promotes the formation of the splay domains within this specific concentration region on top of the molecule's inherent tendency to form polar LC phases.

For binary mixtures of **DIO** with either **PIO** or **CIO,** we find the onset temperature of polar order to vary linearly (to a first approximation). A linear line can be drawn from the N–*N*_X_ transition in **DIO** to the SmA–SmA_F_ transition in **PIO,** passing through the transition to *N*_X_ and SmA_F_ phases across the mixture diagram (Figure 5a blue dashed line). To the authors knowledge, this holds true for all published binary mixtures involving two longitudinally polar LCs.^[^ ^6^, ^16^, ^17^, ^28^, ^29^, ^30^ ^]^ This implies that some temperature exists for a material at which polar ordering of the molecules will be preferable, consistent with some molecular models for the *N*_F_ phase.^[^ ^25^ ^]^ Below this temperature, polar LC phases may be formed seemingly regardless of the underlying preferred LC phase. Multiple factors would influence this temperature, not least combinations of electrostatic and steric contributions.

We suggest that in binary mixtures between LC that shows polar phases and LC that does not (atleast not before crystallizations though we do accept that there will be some LC molecules fundamentally incapable of forming longitudinally polar LC phases), this linear dependence is still true but will be between the apolar–polar transition in the polar LC and a virtual apolar–polar transition in the nonpolar LC. For materials, such as 5CB or E7, this virtual transition seems to be very low and so in binary mixtures the transition to polar order quickly drops out of the phase diagram.^[^ ^28^, ^31^ ^]^ To test this hypothesis, we elected to make mixtures of **DIO** with **CIO** (Figure 6g) and **BIO** (Figure S6, Supporting Information), as the parent molecules did not show any evidence of polar phases. Both materials demonstrated linear dependence on molar concentration for the transition to polar order, enabling a “virtual” transition to a polar phase to be extrapolated which for **BIO** is −1.6 °C and for **CIO** is 1.5 °C. **BIO** shows only N phase and so the phase diagram with **DIO** shows only N‐type phases. The lower‐temperature SmA phase exhibited by **CIO** makes its phase diagram with **DIO** more complex. In high concentrations of either **CIO** or **DIO**, the mixtures behave like the parent molecules, with the expected reduction in transition temperatures as the concentration of the counter molecule is increased. Approaching the eutectic point (40–60 mol% of **DIO**) where the transition to a SmA phase crosses the transition to polar order results in a region where a SmA_F_ phase is formed.

From studying the binary mixtures presented here and elsewhere,^[^ ^16^ ^]^ the empirical linear dependence of the apolar–polar transition for binary mixtures seems to hold regardless of whether it is a polar N phase or a polar Sm phase being formed, that is, there is no apparent interdependence of longitudinal polar and LC order beyond orientational order. This suggests that generally speaking the mechanism that drives the LCs to exhibit longitudinal polar order is at least partially separate to the mechanisms that drive LC to form either N, SmA, or SmC phase. We suggest that “double” transitions (ones where there is a change in LC order AND polar order, i.e., N‐SmA_F_) are improbable for this reason, although there is no a priori reason this should not occur. We do accept that a transition in LC order could occur close enough to a transition in polar order to appear as if it is one transition^[^ ^32^ ^]^ though we suggest that there are separate underlying mechanisms driving each element of this double transition as separate entities. The only requirement of orientational order suggests the possibility of generating polar equivalents of most (if not all) LC phases exhibited by calamitic‐like molecules (e.g., such as SmB or SmG) and indeed recently molecules showing polar SmC phases have been demonstrated.^[^ ^16^ ^]^ Moreover, the interplay between escaping polarity and positional order could be used to generate as yet unseen phase types. This is a significantly different situation from other polar LC phases such as those found in SmC*^[^ ^33^ ^]^ or bent‐core polar LCs^[^ ^34^ ^]^ where the polarity of the system is fundamentally coupled to the more complex LC phase structure and molecular shape and architecture. We stress that this is only the authors’ interpretation of the data. While to the authors’ knowledge there are no binary mixtures between polar LCs that do not show a nonlinear dependence of the apolar–polar transition we do accept that the sample size is relatively small and possibly biased by the fact that generally mixtures are made using extremely chemically similar molecules. We accept and perhaps even expect that for mixtures made between rod‐like longitudinal ferroelectrics and other polar LC architectures, such as polar domes or polar bent‐core LCs, this empirical observation may not hold.

## 3 Conclusion

Several highly polar liquid‐crystalline materials were synthesized as a means to escape the thermal isomerization we observe for the 2,5‐disubstituted 1,3‐dioxane motif in the widely used *N*_F_ host material **DIO**. Although we find only ≈3% of the material isomerizes, this is sufficient to significantly depress phase transitions and presents a significant barrier to working with this material. We developed the material “**PIO**,” finding it to exhibit a SmA_F_ phase. Remarkably, binary mixtures of **PIO** and **DIO** showed an injected antiferroelectric phase (SmA_AF_). Using phase diagrams of **DIO** with **PIO**, **CIO**, and **BIO**, as well as phase diagrams from literature, we show that calamitic LC materials have some temperature at which they will form longitudinal polar LC phases. This apolar–polar transition temperature is independent of the underlying LC phase present as the only requirement for polar order is orientational order which separates the transition into two types: those where LC order changes (e.g., N‐SmA or *N*_F_‐SmA_F_) and those where the polar order changes (e.g., N–*N*_F_ or SmA–SmA_F_).

## Conflict of Interest

The authors declare no conflict of interest.

## Author Contributions

J.H. and C.J.G. contributed equally to this work. C.J.G. and R.J.M. performed chemical synthesis. J.H. and C.J.G. performed microscopy. J.H. performed X‐ray scattering experiments, applied field studies, mixture formulation studies, and DSC analysis. J.H. and R.J.M. performed and evaluated electronic structure calculations. R.J.M. secured funding. The manuscript was written, reviewed, and edited with contributions from all authors.

## Supporting information

Supplementary Material

## Data Availability

The data that support the findings of this study are available in the supplementary material of this article.

## References

[1] T. Geelhaar , K. Griesar , B. Reckmann , Angew. Chem. Int. Ed. 2013, 52, 8798. 10.1002/anie.201301457 23740565

[2] R. J. Mandle , S. J. Cowling , J. W. Goodby , Phys. Chem. Chem. Phys. 2017, 19, 11429. 28422219 10.1039/c7cp00456g

[3] R. J. Mandle , S. J. Cowling , J. W. Goodby , Chem.: Eur. J. 2017, 23, 14554. 28850751 10.1002/chem.201702742 PMC5656819

[4] H. Nishikawa , K. Shiroshita , H. Higuchi , Y. Okumura , Y. Haseba , S. I. Yamamoto , K. Sago , H. Kikuchi , Adv. Mater. 2017, 29, 1702354. 10.1002/adma.201702354 29023971

[5] X. Chen , E. Korblova , D. Dong , X. Wei , R. Shao , L. Radzihovsky , M. A. Glaser , J. E. Maclennan , D. Bedrov , D. M. Walba , N. A. Clark , PNAS 2002, 117, 14021. 10.1073/pnas.2002290117 PMC7322023 32522878

[6] X. Chen , Z. Zhu , M. J. Magrini , E. Korblova , C. S. Park , M. A. Glaser , J. E. Maclennan , D. M. Walba , N. A. Clark , Liq. Cryst. 2022, 49, 1531.

[7] N. Sebastián , M. Čopič , A. Mertelj , Phys. Rev. E 2022, 106, 021001. 36109969 10.1103/PhysRevE.106.021001

[8] N. Sebastián , L. Cmok , R. J. Mandle , M. R. De La Fuente , I. Drevenšek Olenik , M. Čopič , A. Mertelj , Phys. Rev. Lett. 2020, 124, 037801. 32031856 10.1103/PhysRevLett.124.037801

[9] J. Li , H. Nishikawa , J. Kougo , J. Zhou , S. Dai , W. Tang , X. Zhao , Y. Hisai , M. Huang , S. Aya , Sci. Adv. 2021, 7, 5047. 10.1126/sciadv.abf5047 PMC8059932 33883139

[10] R. J. Mandle , N. Sebastián , J. Martinez‐Perdiguero , A. Mertelj , Nat. Commun. 2021, 12, 4962. 34400645 10.1038/s41467-021-25231-0 PMC8367997

[11] A. Mertelj , L. Cmok , N. Sebastián , R. J. Mandle , R. R. Parker , A. C. Whitwood , J. W. Goodby , M. Čopič , Phys. Rev. X 2018, 8, 041025.

[12] P. Kumari , B. Basnet , M. O. Lavrentovich , O. D. Lavrentovich , Science 2024, 383, 1364. 38513040 10.1126/science.adl0834

[13] O. D. Lavrentovich , PNAS 2020, 117, 14629. 32541021 10.1073/pnas.2008947117 PMC7334533

[14] H. Kikuchi , H. Matsukizono , K. Iwamatsu , S. Endo , S. Anan , Y. Okumura , Adv. Sci. 2022, 9, 2202048. 10.1002/advs.202202048 PMC9475520 35869031

[15] X. Chen , V. Martinez , P. Nacke , E. Korblova , A. Manabe , M. Klasen‐Memmer , G. Freychet , M. Zhernenkov , M. A. Glaser , L. Radzihovsky , J. E. Maclennan , D. M. Walba , M. Bremer , F. Giesselmann , N. A. Clark , Proc. Natl. Acad. Sci. 2022, 119, e2210062119. 36375062 10.1073/pnas.2210062119 PMC9704750

[16] C. J. Gibb , J. Hobbs , D. I. Nikolova , T. Raistrick , S. R. Berrow , A. Mertelj , N. Osterman , N. Sebastián , H. F. Gleeson , R. J. Mandle , Nat. Commun. 2024, in press. 10.1038/s41467-024-50230-2 PMC11239904 38992039

[17] C. Yang , F. Ye , X. Huang , J. Li , X. Zhang , Y. Song , S. Aya , M. Huang , Liq. Cryst. 2024, 51, 558.

[18] J. Karcz , J. Herman , N. Rychłowicz , P. Kula , E. Górecka , J. Szydlowska , P. W. Majewski , D. Pociecha , Science 2024, 384, 1096. 38843325 10.1126/science.adn6812

[19] H. Nishikawa , K. Sano , S. Kurihara , G. Watanabe , A. Nihonyanagi , B. Dhara , F. Araoka , Commun. Mater. 2022, 3, 89.

[20] J. Zhou , R. Xia , M. Huang , S. Aya , J. Mater. Chem. C 2022, 10, 8762.

[21] A. E. Kuramshina , V. V. Kuznetsov , Russ. J. Org. Chem. 2010, 46, 871.

[22] C. J. Gibb , R. J. Mandle , J. Mater. Chem. C 2023, 11, 16982.

[23] E. Cruickshank , Chempluschem 2024, 89, e202300726. 38452282 10.1002/cplu.202300726

[24] J. Li , Z. Wang , M. Deng , Y. Zhu , X. Zhang , R. Xia , Y. Song , Y. Hisai , S. Aya , M. Huang , Giant 2022, 11, 100109.

[25] N. V. Madhusudana , Phys. Rev. E 2021, 104, 014704. 34412337 10.1103/PhysRevE.104.014704

[26] J. Szydlowska , P. Majewski , M. Čepič , N. Vaupotič , P. Rybak , C. T. Imrie , R. Walker , E. Cruickshank , J. M. D. Storey , P. Damian , E. Gorecka , Phys. Rev. Lett. 2023, 130, 216802. 37295101 10.1103/PhysRevLett.130.216802

[27] X. Chen , V. Martinez , E. Korblova , G. Freychet , M. Zhernenkov , M. A. Glaser , C. Wang , C. Zhu , L. Radzihovsky , J. E. Maclennan , D. M. Walba , N. A. Clark , PNAS 2023, 120, e2217150120. 36791101 10.1073/pnas.2217150120 PMC9974471

[28] H. Long , J. Li , M. Huang , S. Aya , Liq. Cryst. 2022, 49, 2121.

[29] Y. Song , J. Li , R. Xia , H. Xu , X. Zhang , H. Lei , W. Peng , S. Dai , S. Aya , M. Huang , Phys. Chem. Chem. Phys. 2022, 24, 11536. 35506891 10.1039/d2cp01110g

[30] E. Cruickshank , R. Walker , J. M. D. Storey , C. T. Imrie , RSC Adv. 2022, 12, 29482. 36320775 10.1039/d2ra05628c PMC9562421

[31] K. G. Nazarenko , N. A. Kasian , S. S. Minenko , O. M. Samoilov , V. G. Nazarenko , L. N. Lisetski , I. A. Gvozdovskyy , Liq. Cryst. 2023, 50, 98.

[32] H. Matsukizono , Y. Sakamoto , Y. Okumura , H. Kikuchi , J. Phys. Chem. Lett. 2024, 15, 4212. 38599584 10.1021/acs.jpclett.3c03492 PMC11033931

[33] S. T. Lagerwall , Ferroelectrics 2004, 301, 15.

[34] A. Jákli , O. D. Lavrentovich , J. V. Selinger , Rev. Mod. Phys. 2018, 90, 045004.

