# Supplementary material for: Emergent Antiferroelectric Ordering and the Coupling of Liquid Crystalline and Polar Order
Source: Small Sci. 2024 Jul 3;4(10):2400189. doi: 10.1002/smsc.202400189 (PMC11935032; doi:10.1002/smsc.202400189)
Supplement: Supplementary file 1 — Supplementary Material [file SMSC-4-2400189-s001.pdf]

Emergent Anti-Ferroelectric Ordering and the Coupling of Liquid Crystalline and Polar Order

Supplemental Information

Jordan L. Hobbs * [1], Calum J. Gibb [2], and Richard. J. Mandle* [1,2]

[1] School of Physics and Astronomy, University of Leeds, Leeds, UK, LS2 9JT
[2] School of Chemistry, University of Leeds, Leeds, UK, LS2 9JT

*r.mandle@leeds.ac.uk

Contents: s

1. Experimental Methods
2. Supplementary results
3. Chemical Synthesis and Characterization
4. Supplemental references

1. Experimental Methods

1.1. Chemical Synthesis

Chemicals were purchased from commercial suppliers (Fluorochem, Merck, Ambeed, Specs) and used as received. Solvents were purchased from Merck and used without further purification. Reactions were performed in standard laboratory glassware at ambient temperature and atmosphere and were monitored by TLC with an appropriate eluent and visualised with 254 nm light. Chromatographic purification was performed using a Combiflash NextGen 300+ System (Teledyne Isco) with a silica gel stationary phase and a hexane/ethyl acetate gradient as the mobile phase, with detection made in the 200-800 nm range. Chromatographed materials were subjected to re-crystallisation from an appropriate solvent system.

1.2 Chemical Characterisation Methods

The structures of intermediates and final products were determined using ^{1}H, $^{13}C\{^{1}H\}$, and ^{19}F NMR spectroscopy. NMR was performed using a Bruker Avance III HDNMR spectrometer operating at 400 MHz, 100.5 MHz or 376.4 MHz (^{1}H, $^{13}C\{^{1}H\}$ and ^{19}F, respectively). Unless otherwise stated, spectra were acquired as solutions in deuterated chloroform, coupling constants are quoted in Hz, and chemical shifts are quoted in ppm.

1.3 Mesophase Characterisation

Transition temperatures and measurement of associated latent heats were measured by differential scanning calorimetry (DSC) using a TA instruments Q2000 heat flux calorimeter with a liquid nitrogen cooling system for temperature control. Between 3-8 mg of sample was placed into T-zero aluminium DSC pans and then sealed. Samples were measured under a nitrogen atmosphere with 10 °C min^{-1} heating and cooling rates. The transition temperatures

and enthalpy values reported are averages obtained for duplicate runs. In general LC phase transition temperatures are measured on cooling from the onset of the transition while melt temperatures were measured on heating to avoid crystallization loops that can occur on cooling. Phase identification by polarised optical microscopy (POM) was performed using a Leica DM 2700 P polarised optical microscope equipped with a Linkam TMS 92 heating stage. Samples were studied sandwiched between two untreated glass coverslips.

1.4 DFT Calculations

Electronic structure calculations were performed using Gaussian G16 revision C.02 [1] and with a B3LYP-GD3BJ/cc-pVTZ [2–4] basis set. For each input structure we used the ETKDGv3 rules-based method [5] to generate the lowest energy conformer for a given chemical structure. The conformer then underwent geometry optimisation followed by a frequency calculation to confirm the geometry to be at a minimum.

To obtain the optimised geometry of the various conformers of **DIO** studied here we took the lowest energy conformer which was equatorial trans **DIO** (this is "standard" **DIO**) and altered the structure of the dioxane ring to obtain both axial-trans **DIO** and the stable intermediate twist-boat state between the trans axial and equatorial forms. Both were verified as a minimum from frequency calculations. Transition states were found from estimating the "likely" medium position between the initial and final states and then optimising. The force constants were calculated at the beginning of the optimisation step and then not recalculated. The molecule was verified as a transition state from frequency calculations where only a single imaginary frequency was observed. It was further confirmed that this was the correct transition state by visualizing the vibration associated with the imaginary frequency and then calculating the intrinsic reaction coordinate (IRC) pathway and visually verifying that the start and end points correspond to the correct structures. For the IRC calculations the force constants were computed for the initial point only. The local quadratic approximation was used for the predictor step and 150 points were calculated along the reaction path in each direction.

Electrostatic potential (ESP) surfaces were calculated by using the *formchk* and *cubegen* utilities. Both the electron density and ESP cube files were calculated using "fine" data resolution. The ESP surface is displayed at an electron density iso-surface of 0.0004. The 3D data was reduced into 1D by taking each plane across the z-axis given in the cube file, finding the isoline contour across each plane where the electron density equals 0.0004 (to reflect the iso-surface), and then averaging all ESP values that lie along that loop. We justify this averaging step through the assumption that the molecule will experience nearly free rotation around it's z-axis. A further step of rescaling the values obtained by the length of the contour allows to account for the fact that at the molecular extremes the values are distorted by the reducing molecular volume. This final step effectively gives the ESP as electric flux i.e. the strength of the electric field due to the molecular dipole through the contour.

1.5 X-ray Scattering

X-ray scattering measurements, both small angle (SAXS) and wide angle (WAXS) where recorded using an Anton Paar SAXSpoint 5.0 beamline machine. This was equipped with a primux 100 Cu X-ray source with a 2D EIGER2 R detector. The X-rays had a wavelength of 0.154 nm. Samples were filled into thin-walled quartz capillaries 1 mm thick. Temperature was controlled using an Anton Paar heated sampler with a range of -10 ℃ to 110 ℃ and the

samples held in a chamber with an atmospheric pressure of <1 mBar. Samples were held at 110 ℃ to allow for temperature equilibration across the sample and then slowly cooled while stopping to record the scattering patterns.

No external alignment technique was used and so these measurements should be considered as “powder” samples. It should be noted that some spontaneous alignment of the LCs within the capillaries did occur leading to the classic “lobe” pattern seen in the 2D patterns. 1D patterns were obtained by radially integrating the 2D SAXS patterns. Peak position and FWHM was recorded and then converted into d spacing following Bragg’s law.

1.6 Measurement of Spontaneous Polarization (P_S)

Spontaneous polarisation measurements are undertaken using the current reversal technique [6,7]. Triangular waveform AC voltages are applied to the sample cells with an Agilent 33220A signal generator (Keysight Technologies), and the resulting current outflow is passed through a current-to-voltage amplifier and recorded on a RIGOL DHO4204 high-resolution oscilloscope (Telonic Instruments Ltd, UK). Heating and cooling of the samples during these measurements is achieved with an Instec HCS402 hot stage controlled to 10 mK stability by an Instec mK1000 temperature controller. The LC samples are held in 4μm thick cells with no alignment layer, supplied by Instec. The measurements consist of cooling the sample at a rate of 1 Kmin^{-1} and applying a set voltage at a frequency of 10 Hz. The voltage was set such that it would saturate the measured P_S and was determined before final data collection.

There are three contributions to the measured current trace: accumulation of charge in the cell (I_c), ion flow (I_i), and the current flow due to polarisation reversal (I_p). To obtain a P_S value, we extract the latter, which manifests as one or multiple peaks in the current flow, and integrate as:

$$P_S = \int \frac{I_p}{2A} dt \qquad \textbf{(2)}$$

where A is the active electrode area of the sample cell. For the N, N_X and, to a lesser extent, the N_F phase, significant amounts of ion flow is present. For materials and mixtures that showed a paraelectric N phase followed by the anti-ferroelectric N_X phases, the N phase always showed some pre-transitional polarisation as well as the significant ion flow mentioned previously. Since the following phase was anti-ferroelectric, this pre-transitional polarisation was anti-ferroelectric in character and so was decoupled from the ion flow in the same way as the N_X phase and as such the P_S of the N and N_X phases was obtained by integrating the peak least affected by ion flow and then doubled to get the total area under both peaks [8]. For the SmA_F phase found in these materials generally we observed low charge accumulation and ion flow allowing for the baseline to be easily defined and the integrated area of the peak or peaks to be obtained accurately.

[6,7] [8]

2. Supplemental results

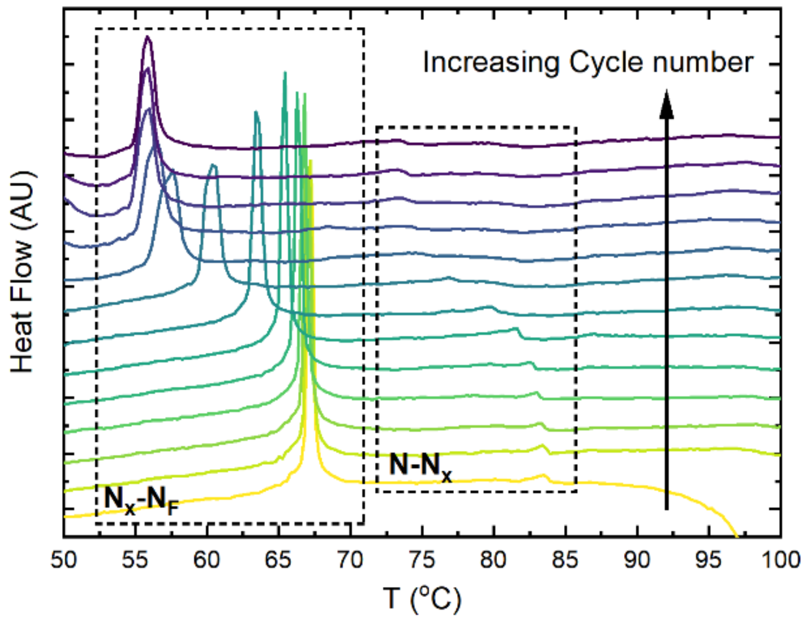

Fig. S1 DSC thermograms of **DIO** where for each successive run the upper temperature of the run was increased by 10 °C. The direction of the arrow and colour gradient of the DSC cycles (yellow to purple) show the direction of increasing cycle number. The cycles are measured from an upper T of 100 °C to 220 °C showing the step like behaviour of the transitions due to *equatorial trans* to *axial trans* isomerisation.

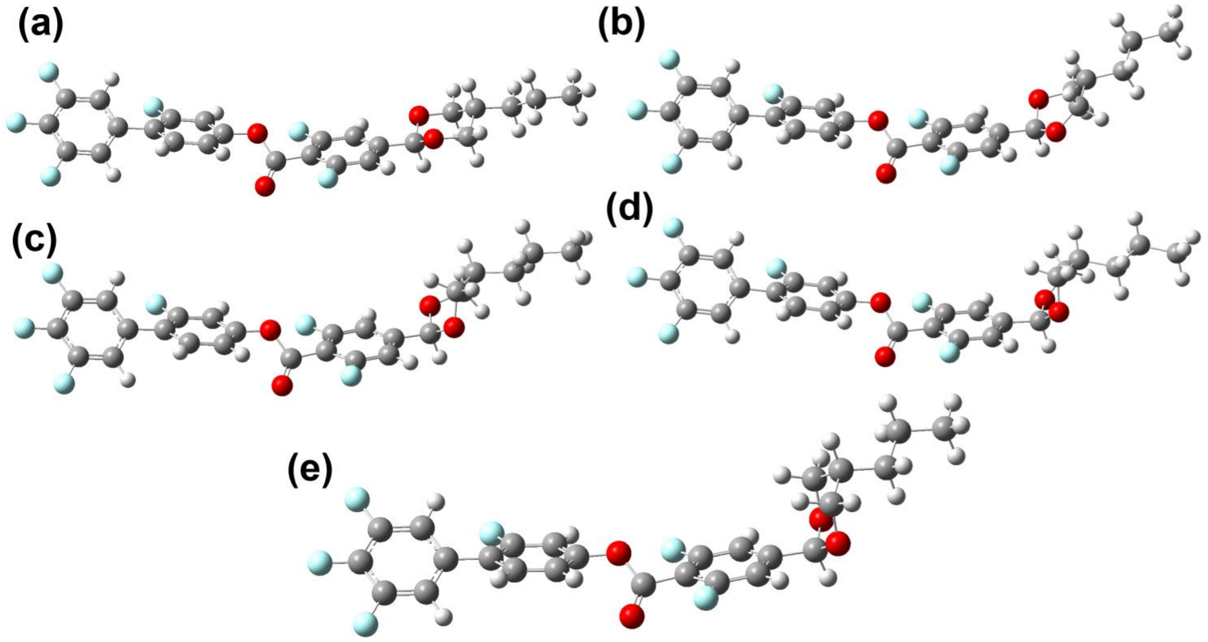

Fig. S2 DFT optimised structures of **DIO** in the a) *equatorial trans* (eq-trans), b) 1st half-chair transition state (TS1), c) twist boat (TB), d) 2nd half-chair transition state (TS2) and e) *axial trans* (ax-trans) forms. All calculations conducted at B3LYP-GD3BJ/aug-cc-pVTZ level of DFT.

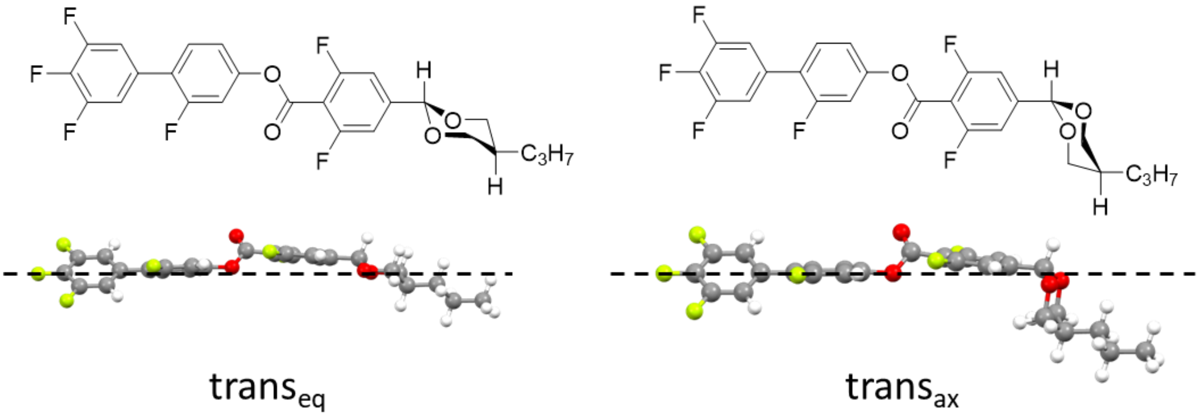

Fig. S3 Chemical structures and DFT optimised ground states of equatorial and axial trans **DIO;** the significant deviation from linearity leads to the observed reduction in transition temperatures.

Table S1. Transition temps etc

	K		SmA_F-SmA		SmA-N		N-Iso	
	T (ºC)	ΔH (kJ mol^{-1})	T (ºC)	ΔH (kJ mol^{-1})	T (ºC)	ΔH (kJ mol^{-1})	T (ºC)	ΔH (kJ mol^{-1})
**1**	74.0	30.0	-	-	-	-	-	-
**2**	85.3	25.4	-	-	-	-	-	-
**3**	107.3	30.0	-	-	-	-	[71.4]	0.3
**4**	101.6	34.2	-	-	-	-	-	-
**5**	102.7	23.4	-	-	-	-	[93.3]	0.3
**6**	45.9	14.7	-	-	-	-	66.5	0.3
**7**	72.1	17.2	-	-	-	-	227.5	0.6
**8**	130.0	26.7	-	-	-	-	240.4	0.6
**9**	94.3	22.2	-	-	-	-	140.4	0.4
**10**	88.1	25.1	-	-	[59.3]	0.05	208.7	0.6
**11**	98.8	23.7	70.0†	N/A	115.8	0.2	201.6	0.5

† determined by POM studied, no enthalpy given; '[ ]' indicates a monotropic phase transition.

Table S2. DFT parameters

	Dipole (D)	Dipole Angle (º)	Length (Å)	Width (Å)	Aspect Ratio
**1**	7.06	12.8	19.03	5.09	3.74
**2**	6.99	5.9	18.91	4.71	4.02
**3**	6.36	13.2	18.88	4.89	3.86
**4**	5.82	12.9	18.64	5.28	3.53
**5**	5.27	13.4	18.57	5.56	3.34
**6**	5.21	12.8	18.88	4.91	5.21
**7**	5.26	16.1	23.92	5.39	5.26
**8**	6.68	9.6	23.03	6.06	6.68
**9**	10.04	9.5	23.61	5.49	10.04
**10**	7.48	10.5	23.12	6.08	7.48
**11**	7.97	10.2	23.09	5.51	7.97

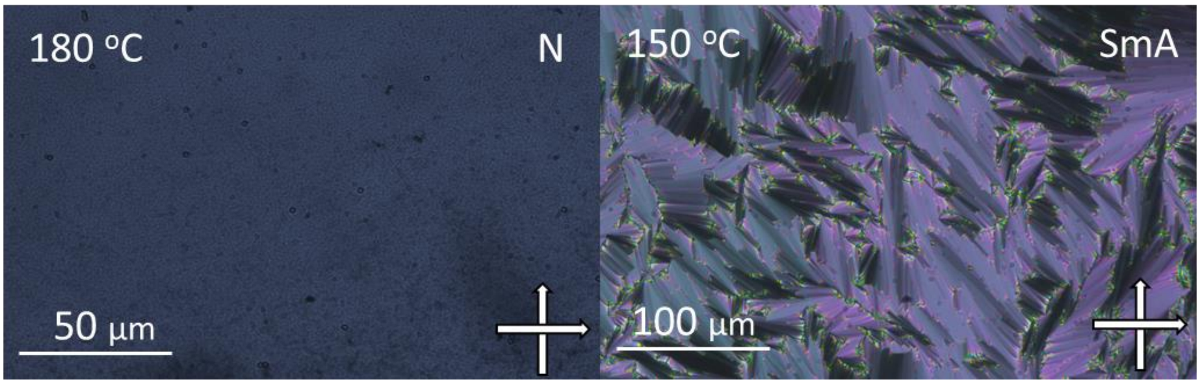

Fig. S4 POM micrographs of a planar texture (left) and focal conic fan texture (right) of the N and SmA phases respectively, observed for **10** (**CIO**) observed within a 5 µm cell with no anchoring condition.

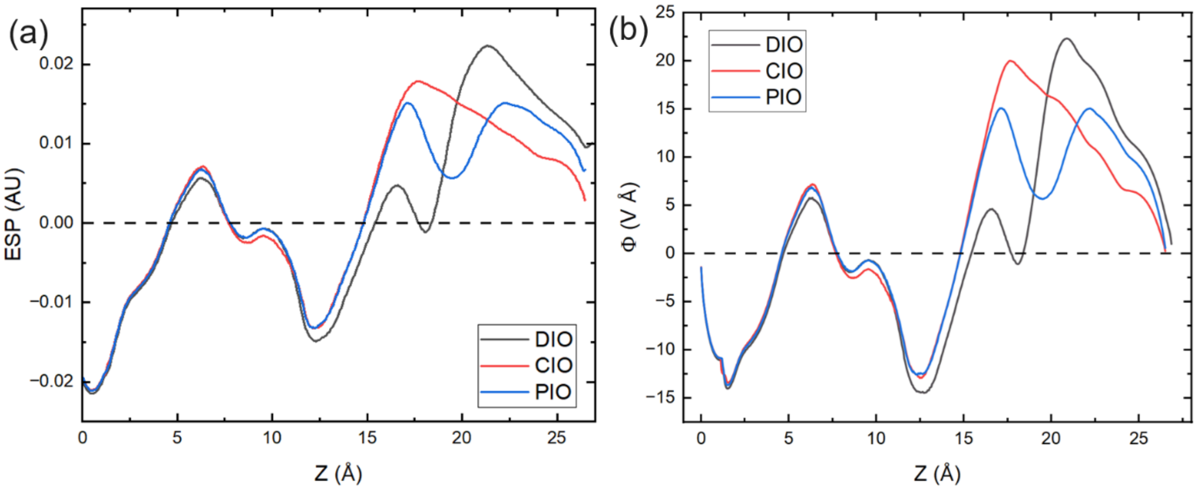

Fig. S5. a) Electrostatic potential along the z-axis of the molecules **DIO**, **CIO** and **PIO**. AU stands for atomic units. b) the electrostatic potential along the z-axis rescaled by the length of the iso-density value contour around the molecule at the specific z point.

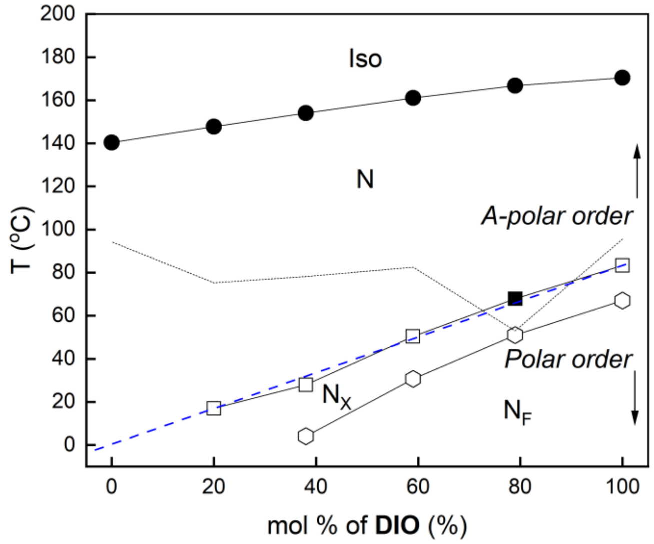

Fig. S6. Phase diagram of binary mixtures of **DIO** and **9** (**BIO**). Blue dashed line indicates the linear extrapolation of the apolar-polar transition giving a virtual apolar-polar transition temperature of -1.6 °C

3. Chemical Synthesis and Structural Characterization

3.1 Overall reaction scheme

We synthesised 2’,3,4,5-tetrafluoro-4’-hydroxybiphenyl *via* the Suzuki-Miyaura cross-coupling of 4-bromo-3-fluorophenol with 3,4,5-trifluorobenzene boronic acid with $Pd(OAc)_2$/SPHOS as the catalyst, affording the title compound in 94% yield on ~ 100 mmol scale (28 g). Subsequent esterification (using EDC.HCl and DMAP) with a selection of carboxylic acids, some of which were synthesised as described below (***i1-i6***) and others which were available in house ***(i7-i11),*** afforded the target DIO-homologues detailed in Table 1. For in-house prepared carboxylic acids, iron catalysed Kumada cross coupling of propyl magnesium bromide with either methyl 4-chloro-2-fluorobenzoate or methyl 4-chloro-2,6-difluorobenzoate, followed by basic hydrolysis and acid workup, afforded ***i1*** and ***i12;*** lithiation/carboxylation of *trans* 5-(4-propylcyclohexyl)-1,3-difluorobenzene afforded ***i3***; condensation of 4-borono-2,6-difluorobenzoic acid with 2-propylpropan-1,3-diol in THF with 4A molecular sieves afforded ***i4***; Suzuki-Miyaura cross coupling of 4-bromopropylbenzene with either 3-fluorobenzene boronic acid or 3,5-difluorobenzene boronic acid, followed by lithiation/carboxlyation and acidic workup, afforded ***i5*** and ***i6***.

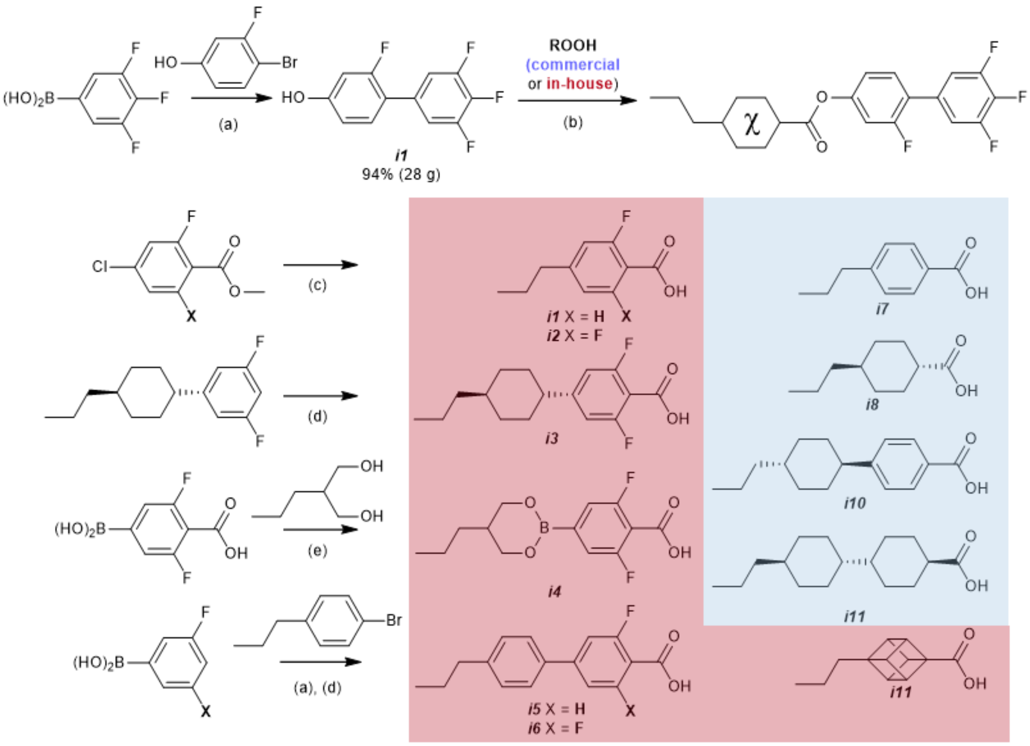

(a) $Pd(OAc)_2$, SPHOS, K_2CO_3, THF / H_2O, N_2, 60 °C;
(b) EDC.HCl, DMAP, DCM;
(c) i) PrMgCl, $Fe(acac)_3$, THF / NMP, N_2; ii) KOH, H_2O/EtOH; iii) HCl, H_2O;
(d) i) nBuLi, THF, -78 °C, N_2; ii) CO_2, - 78 °C; iii) HCl, H_2O, 0 °C;
(e) THF, 4Å MS, 50°C, N_2;

Scheme S1. Synthetic scheme for synthesis of compounds **1-11** described in this work. Carboxylic acids were either obtained commercially (blue) or synthesised in house (red).

3.2 Synthesis of chemical intermediates (*i1-i8*)

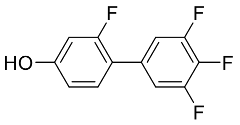

2’,3,4,5-tetrafluoro-4’-hydroxybiphenyl (i1)

A solution of 4-bromo-2-fluorophenol (23.5 g, 0.123 mol) in biphasic mixture of THF (125 ml) and 2M aqueous K_2CO_3 (100 ml) was degassed by sparging with argon for 15 minutes. The Pd-SPHOS catalyst was prepared by degassing 5 ml of THF with argon sparging for 5 minutes, adding solid $Pd(OAc)_2$ (50 mg) and SPHOS (100 mg) and stirring for 5 minutes while sparging with argon. The biphasic reaction mixture was heated to reflux under an argon atmosphere. 3,4,5-Trifluorobenzeneboronic acid (25 g, 0.144 mol) was added as a solid in one portion, followed by the Pd-SPHOS catalyst as a solution in THF. The reaction mixture was heated under reflux with vigorous stirring, under an atmosphere of dry argon, for 1 hour at which point TLC analysis showed total consumption of the starting phenol. The solution was cooled, the aqueous layer separated and washed with ethyl acetate (3x 50 ml), and discarded. The combined organics were sequentially washed with saturated aqueous ammonium chloride (50 ml), brine (50 ml). The organics were then dried over $MgSO_4$, filtered, and volatiles removed *in vacuo*. The crude material was filtered over a short plug of silica gel, eluting with DCM, and

then recrystalised from ethanol, affording the title compound as an amorphous white solid. Spectral data matched an authentic sampled purchased from Manchester Organics.

Yield: 28.0 g (94%)

Rf (DCM): 0.22

^{1}H NMR (400 MHz): 7.23-7.30 (1H, m, Ar-**H**), 7.10-7.20 (2H, m, Ar-**H**), 6.68 – 6.75 (2H, m, Ar-**H**), 5.31 (1H, s, ArO**H**).

^{19}F NMR (376.4 MHz): -115.37 (1F, t, J_{H-F} = 10.3 Hz, Ar-**F**), -134.77 (2F, *dd*, J_{F-F} = 20.6 Hz, J_{H-F} = 9.0 Hz, Ar-**F**), -162.48 (1F, tt, J_{F-F} = 20.6 Hz, J_{H-F} = 6.5 Hz, Ar-**F**).

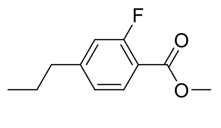

2-fluoro-4-propyl propylbenzoate (i2)

Methyl 4-chloro-2-fluorobenzoate (3 g, 16.8mmol) and Fe(acac)$_3$ (352 mg, 1 mmol) were dissolved into in anhydrous THF (40 ml) and anhydrous NMP (4 ml) with stirring, under an atmosphere of dry nitrogen gas. Once homogenous, propyl magnesium bromide (1 M, 25ml, 25 mmol) in 2-MeTHF was added dropwise at ambient temperature, prompting a colour change from red to green then brown and finally black. The solution was stirred for approximately 1 hour, at which point the colour returned to red/brown. TLC showed consumption of the starting material (Rf = 0.88, DCM) and the formation of a new spot (Rf = 0.84, DCM). The reaction was quenched with saturated aqueous $NHCl_4$ (50 ml). The organic layer was separated and retained, the aqueous was washed with ethyl acetate (3x 20 ml) and discarded. The organic layer was washed with water (5x 50 ml) and finally brine (1x 100 ml). The organic layer was then dried over $MgSO_4$, concentrated to dryness, and trace NMP removed with high vacuum. Purification with flash chromatography over silica (RediSep 40 g) with a gradient of hexane/EtOAc afforded methyl 2-fluoro-4-propylbenzoate as a colourless oil.

Yield: 2.9 g, 94 %

R_f (DCM): 0.84

^{1}H NMR (400 MHz): 7.83 (1H, t, J = 7.8 Hz, Ar-**H**), 6.99 (1H, dd, J = 7.7 Hz, J = 2.0 Hz, Ar-**H**), 6.93 (1H, dd, J = 11.9 Hz, J = 1.0 Hz, Ar-**H**), 3.90 (3H, s, COOC-**H$_3$**), 2.60 (2H, t, J = 7.0 Hz, Ar-C**H$_2$**-CH$_2$), 1.63 (2H, m, CH$_2$-C**H$_2$**-CH$_3$), 0.92 (3H, t, J = 7.0 Hz, CH$_2$-C**H$_3$**)

^{19}F NMR (376 MHz): 110.23 (1F, dd, *J* = 7.7 Hz, *J* = 11.9 Hz, Ar-**F**)

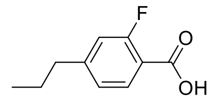

2-Fluoro-4-propylbenzoic acid (i3)

Methyl 2-fluoro-4-propylbenzoate (***i1*** , 2.9 g, 16.6 mmol) was dissolved into ethanol (50 ml) and heated under reflux with stirring. A solution of aqueous 2M NaOH (50 ml) was added, and the solution heated under reflux for 18 h. TLC showed complete consumption of the starting material (R_f = 0.84, DCM) and formation of a new spot (Rf = 0.0, DCM). The reaction solution was cooled to ambient temperature and acidified with 2M HCl, affording a white precipitate. The precipitated was collected by filtration, washed with cold water (2x 20 ml), and finally recrystalised from ethanol (-20 °C) to afford the title compound as colourless needles.

Yield 2.5 g (93 %; colourless needles)

1H NMR (400 MHz): 7.96 (1H, t, J = 7.9 Hz, Ar-**H**), 7.07 (1H, ddd, J = 8.1Hz, J = 1.5 Hz, Ar-**H**), 7.01 (1H, dd, J = 12.0 Hz, J = 1.5 Hz, Ar-**H**), 2.67 (2H, t, J = 7.0 Hz, Ar-C$\mathbf{H_2}$-CH_2), 1.70 (2H, m, CH_2-C$\mathbf{H_2}$-CH_3), 0.98 (3H, t, J = 7.4 Hz, CH_2-C$\mathbf{H_3}$).

^{19}F NMR (376 MHz): -109.04 (1F, dd, *J* = 7.7 Hz, *J* = 12.0 Hz, Ar-**F**)

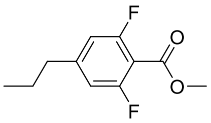

2,6-Difluoro-4-propyl propylbenzoate (i4)

Quantities used: methyl 2,6-difluoro-4-chlorobenzoate (1.00 g, 4.84 mmol), propyl magnesium chloride (1M in 2-MeTHF, 6.00 mmol, 6.00 ml), Fe(acac)3 (0.35 g, 1.00 mmol), THF (20.0 ml) NMP (2.00 ml). The experimental procedure was as described for (***i1***). Purification *via* column chromatography with a gradient of hexane/EtOAc over silica afforded the title compound as a colorless oil, used directly in the saponification step described for ***i5*** without analysis.

Yield: 0.83g, 80%

R_f(DCM): 0.52

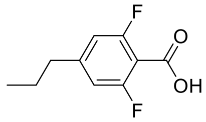

2,6-Difluoro-4-propylbenzoic acid (i5)

Quantities used: methyl 2,6-difluoro-4-propylbenzoate (0.83 g, 3.87 mmol), ethanol (20.0 mL), 2M aqueous sodium hydroxide (10 mL). The reaction procedure was as described for the synthesis of ***I-3***, affording the title compound as colourless needles.

Yield: 0.69 g, 89%

^{1}H NMR (400 MHz, CDCl3) δ: 6.73 (d, J = 10.2 Hz, 2H, Ar-**H**), 2.53 (t, J = 7.6 Hz, 2H, Ar-C$\mathbf{H_2}$-CH_2), 1.57 (h, J = 7.5 Hz, 2H, Ch2-C$\mathbf{H_2}$-CH_3), 0.87 (t, J = 7.4 Hz, 3H, CH_2-C$\mathbf{H_3}$).

^{19}F NMR (376 MHz, $CDCl_3$) δ -108.74 (d, *J* = 10.7 Hz, Ar-**F**).

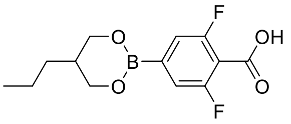

2-(3,5-difluoro-4-carboxyphenyl)-5-propyl-[1,3,2]dioxaborinane (i6)

A suspension of 4-borono-2,6-difluorobenzoic acid (1g, 7.58 mmol), 2-propylpropan-1,3-diol (0.91 g, 7.8 mmol) and dried 4Å molecular sieves (1 g) in dry THF (10 ml) was heated with vigorous stirring under an atmosphere of dry nitrogen gas for 24 h. TLC showed complete consumption of the starting material (R_F EtOAc = 0.3) and formation of a new spot (R_F EtOAc ~ 0.5). The volatiles were removed under reduced pressure. The crude material was dissolved into acetone (~ 5 ml), cooled to -20 dC, and precipitated *via* trituration with cold (-20 °C) hexane to afford the title compound as an off-white solid.

Yield: 1.7 g (79%)

Rf (EtOAc): 0.5

^{1}H NMR (400 MHz): 7.24 (2H, d, *J* = 9.3 Hz, Ar$\underline{H}$), 4.09 (2H, *J* = 4.4 Hz, *J* = 11.0 Hz, B-[OCH$\underline{H}_{ax}$CH-CH$\underline{H}_{ax}$-O]), 3.69 (2H, *J* = 4.4 Hz, *J* = 11.0 Hz, B-[OCH$\underline{H}_{eq}$-CH-CH$\underline{H}_{eq}$-O]), 2.04 (1H, ttt, *J* = 2.0 Hz, *J* = 4.9 Hz, *J* = 8.8 Hz, B-[OCH$_2$-C$\underline{H}$(-CH$_2$-CH$_2$...)-CH$_2$-O]), 1.26-1.38 (2H, m, CH-CH$_2$-C$\underline{H}_2$-CH$_3$), 1.15-1.24 (2H, m, CH-C$\underline{H}_2$-CH$_2$), 0.88 (3H, t, *J* = 7.2 Hz, Ar-CH$_2$-CH$_2$-C**H$_3$**),

^{19}F NMR (376 MHz): -110.78 (2F, S, Ar$\underline{F}$)

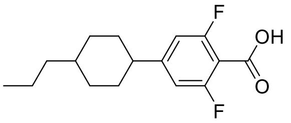

trans 4-(4-propylcyclohexyl)-2,6-difluorobenzoic acid (i7)

A solution of n-butyl lithium (1.6 M in hexanes, 7.50 mL, 12.0 mmol) was added dropwise to a stirred, cooled (-78 °C) solution of *trans* 4-(4-propylcyclohexyl)-2,6-difluorobenzene in anhydrous THF (10 mL) under an atmosphere of dry nitrogen. The aryl lithum solution was allowed to stir for 30 minutes before adding solid carbon dioxide (~ 2.0 g) in a single portion with vigorous stirring and allowing too slowly warm to ambient temperature (~ 2 h). The basic solution was acidified with 2M HCl (~ 50 ml) and extracted with EtOAc. The organics were then dried over $MgSO_4$ and a white solid retrieved under reduced pressure. These were then purified by recrystallization from EtOH/Hexane to give white needles.

Yield: 1.7 g, 60%

R_f (EtOAc): 0.42

^{1}H NMR (400 MHz, CDCl$_3$) δ 11.96 (s, 1H, Ar-O**H**), 6.97 – 6.69 (m, 2H, Ar-**H**), 2.59 – 2.37 (m, 1H, Ar-C**H**-(CH$_2$)$_2$), 1.98 – 1.80 (m, 4H, CH-C**H$_2$**-CH$_2$ and Ar-CH(C**H$_{eq}$**)H$_{ax}$ x2)*, 1.47 – 0.96 (m, 10H, CH$_2$(cyclohexane) and CH$_2$-C**H$_2$**-CH$_3$)*, 0.89 (t, *J* = 7.2 Hz, 3H, CH$_2$-C**H$_3$**).

^{19}F NMR (376 MHz, $CDCl_3$) δ -108.39 (d, *J* = 10.6 Hz, Ar-F).

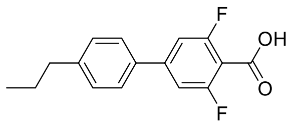

(4'-propyl-3,5-difluorobiphenyl-4-carboxylic acid) (i8)

A solution of n-butyl lithium (1.6 M in hexanes, 31.0 mL, 51.0 mmol) was added dropwise to a stirred, cooled (-78 °C) solution of *trans* 4-(4-propylphenyl)-2,6-difluorobenzene (9.79 g, 42.0mmol) in anhydrous THF (10 mL) under an atmosphere of dry nitrogen. The aryl lithium solution was allowed to stir for 30 minutes at this same temperature before adding solid carbon dioxide (~ 2.0 g) in a single portion with vigorous stirring. The solution was then allowed too slowly warm to ambient temperature (~ 2 h). The basic solution was acidified with 2M HCl (~ 50 ml) and extracted with EtOAc. The organics were then dried over $MgSO_4$ and a white solid retrieved under reduced pressure. These were then purified by recrystallization from EtOH/Hexane to give white needles.

Yield: 9.50 g, 81%

R_f (EtOAc): 0.37

^{1}H NMR (400 MHz, CDCl3) δ 7.53 (ddd, J = 8.2, 2.3, 1.8 Hz, 2H, Ar-**H**), 7.32 (ddd, J = 8.2, 1.6, 1.4 Hz, 2H, Ar-**H**), 7.28 – 7.18 (m, 2H, Ar-**H**), 2.67 (t, J = 7.3 Hz, 2H, Ar-C**H**$_{2}$-CH_2), 1.71 (h, J = 7.5 Hz, 2H, CH_2-C**H**$_{2}$-CH_3), 1.00 (t, J = 7.3 Hz, 3H, CH_2-C**H**$_{3}$).

^{19}F NMR (376 MHz, $CDCl_3$) δ -107.65 (d, *J* = 10.6 Hz, Ar-**F**).

3.3 Characterisation data for final LC compounds

3.3.1 General Steglich Esterification Procedure

Unless otherwise noted, a round bottomed flask or 14 ml vial was charged with carboxylic acid (1 mmol, 1 eqv.), phenol (1 mmol, 1 eqv.), EDC.HCL (1.5 mmol, 1.5 eqv.) and DMAP (< 5 mg). A stirrer bar was added, and sufficient DCM added to ensure complete solvation (4 – 50 ml). The reaction vessel was closed with a stopper or cap and stirred until complete consumption of either the acid or phenol as judged by TLC analysis. Once complete, the reaction solution was concentrated and purified by flash chromatography over silica gel with a gradient of hexane/EtOAc using a Combiflash NextGen300+ system. The chromatographed material was dissolved into the minimum quantity of DCM, filtered through a 0.2 micron PTFE filter, concentrated to dryness and finally recrystalised from the indicated solvent system.

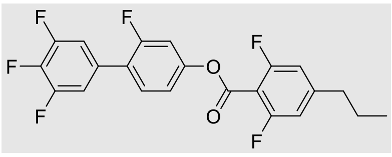

(**1**)

Yield: 326 mg (77 %, colourless crystals);

R_f (DCM): 0.89

^{1}H NMR (400 MHz): δ 7.43 (td, *J* = 8.7, 4.3 Hz, 1H, Ar-**H**), 7.23 – 7.07 (m, 6H, Ar-**H**), 6.86 (d, *J* = 10.1 Hz, 2H, Ar-**H**), 2.65 (t, *J* = 7.6 Hz, 2H, Ar-C**H₂**-CH₂), 1.68 (h, *J* = 7.4 Hz, 2H, CH₂-C**H₂**-CH₃), 0.97 (t, *J* = 7.3 Hz, 3H, CH₂-C**H₃**).

^{13}C{^{1}H} NMR (101 MHz, $CDCl_3$): 164.76, 160.66, 158.17, 152.49, 151.92, 151.82, 149.96, 149.68, 140.73, 138.22, 131.02, 130.57, 130.53, 130.36, 128.87, 126.32, 123.82, 123.69, 118.36, 118.32, 113.32, 113.28, 113.10, 113.06, 110.92, 110.66, 38.15, 24.25, 13.75.

^{19}F NMR (376 MHz): δ -106.69 (d, *J* = 9.0 Hz, Ar-**F**), -109.44 (d, *J* = 10.3 Hz, Ar-**F**), -114.07 – -115.90 (m, Ar-**F**), -134.14 (ddd, *J* = 40.1, 20.7, 8.9 Hz, Ar-**F**), -161.21 (tt, *J* = 20.5, 6.7 Hz, Ar-**F**).

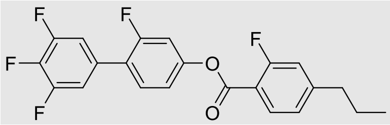

(**2**)

Yield: 332 mg (82 %, colourless crystals);

R_f (DCM): 0.84

^{1}H NMR (400 MHz): 8.01 (t, *J* = 7.8 Hz, 1H, Ar-**H**), 7.42 (t, *J* = 8.7 Hz, 1H, Ar-**H**), 7.25 – 6.98 (m, 6H, Ar-**H**), 2.68 (t, *J* = 6.8 Hz, 2H, Ar-C**H₂**-CH₂), 1.70 (h, *J* = 7.4 Hz, 2H, CH₂-C**H₂**-CH₃), 0.98 (t, *J* = 7.3 Hz, 3H, CH₂-C**H₃**).

^{13}C{^{1}H} NMR (101 MHz, $CDCl_3$) [reported peaks]: 163.81, 162.31, 162.27, 161.21, 160.63, 158.14, 152.45, 152.35, 152.27, 151.52, 151.41, 150.01, 149.90, 140.75, 138.23, 132.41, 130.97, 130.58, 130.54, 124.53, 124.50, 123.96, 123.84, 118.32, 118.28, 117.18, 116.96, 114.66, 114.57, 113.33, 113.29, 113.24, 113.11, 113.07, 110.88, 110.63, 37.86, 23.88, 13.66.

^{19}F NMR (376 MHz): -108.31 (dd, *J* = 11.9, 7.5 Hz, Ar-**F**), -114.66 (t, *J* = 9.9 Hz, Ar-**F**), -134.25 (dd, *J* = 20.6, 8.8 Hz, Ar-**F**), -161.31 (tt, *J* = 20.7, 6.6 Hz), Ar-**F**.

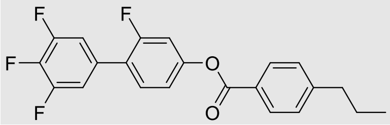

(**3**)

Yield: 0.275 mg (72 %, colourless crystals);

R_f (DCM): 0.83

^{1}H NMR (400 MHz): 8.11 (ddd, *J* = 8.3, 1.9, 1.8 Hz, 2H, Ar-**H**), 7.43 (t, *J* = 8.6 Hz, 1H, Ar-**H**), 7.34 (ddd, *J* = 8.5, 1.9, 1.8 Hz, 2H, Ar-**H**), 7.23 – 7.16 (m, 2H, Ar-**H**), 7.14 (t, *J* = 2.8 Hz, 1H, Ar-**H**), 7.13 – 7.09 (m, 1H, Ar-**H**), 2.70 (t, *J* = 6.8 Hz, 2H, Ar-C**H₂**-CH₂), 1.70 (h, *J* = 7.5 Hz, 2H, CH₂-C**H₂**-CH₃), 0.98 (t, *J* = 7.4 Hz, 3H, CH₂-C**H₃**).

^{13}C{^{1}H} NMR (101 MHz, $CDCl_3$) [reported peaks]: 164.76, 160.66, 158.17, 152.49, 151.92, 151.82, 149.96, 149.68, 140.73, 138.22, 131.02, 130.57, 130.53, 130.36, 128.87, 126.32,

123.82, 123.69, 118.36, 118.32, 113.32, 113.28, 113.10, 113.06, 110.92, 110.66, 77.35, 38.15, 24.25, 13.75.

^{19}F NMR (376 MHz): -114.72 (t, *J* = 9.7 Hz, Ar-**F**), -134.26 (dd, *J* = 20.5, 8.8 Hz, Ar-**F**), -161.35 (tt, *J* = 20.7, 6.6 Hz, Ar-**F**).

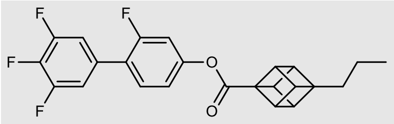

(4)

Yield: 152 mg (78 %; colourless prisms)

R_f (DCM): 0.91

^{1}H NMR: 7.36-7.26 (1H, m, Ar**H**), 7.14-7.04 (2H, m, Ar**H**), 6.99-6.91 (2H, m, Ar**H**), 4.22-4.14 (3H, m, Cub**H**), 3.78-3.72 (3H, m, Cub**H**), 1.57-1.47 (2H, m, Cub-C**H**$_2$-CH$_2$-CH$_3$), 1.36-1.23 (2H, m, Cub-CH$_2$-C**H**$_2$-CH$_3$), 0.88 (3H, t, *J* = 7.4 Hz, Cub-CH$_2$-CH$_2$-C**H**$_3$)

^{13}C{^{1}H} NMR: 170.37, 160.60, 158.10, 152.6-152.2 (m), 151.66 (m), 140.41 (m), 130.44 (d, *J* = 4.1 Hz), 123.31 (d, *J* = 15.4 Hz), 118.18 (d, *J* = 3.7 Hz), 113.61-112.87 (m), 110.59 (d, *J* = 25.7 Hz), 60.01, 56.13, 46.38, 46.30, 37.41, 17.53, 14.20

^{19}F NMR: -114.92 (1F, t, *J* = 9.9 Hz, Ar**F**), -134.36 (2F, dd, *J* = 8.7 Hz, *J* = 20.5 Hz, Ar**F**), -161.47 (1F, tt, *J* = 6.5 Hz, *J* = 20.5 Hz, Ar**F**)

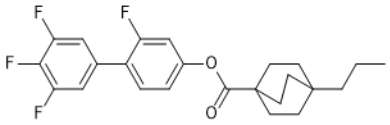

(5)

Yield: 161 mg (77%, colurless needles)

$R_{f\,(DCM)}$: 0.95

^{1}H NMR: 7.32-7.24 (1H, m, ArH), 7.14-7.01 (2H, m, ArH), 6.90-6.82 (2H, m, ArH), 1.89-1.81 (6H, m, BCOH), 1.43-1.35 (6H, m, BCOH), 1.25-1.08 (2H, m, -CH_2-CH_2-CH_3), 1.08-0.98 (2H, m, -CH_2-CH_2-CH_3), 0.81 (3H, t, *J* = 7.7 Hz, (2H, m, -CH_2-CH_2-CH_3)

^{13}C{^{1}H} NMR: 176.29, 160.58, 159.09, 151.91 (d, *J* =12.0 Hz), 151.14 (dd, *J* = 250.0 Hz, *J* = 9.8 Jz), 140.63 (m), 138.31 (m), 130.40 (d, *J* = 4.0 Hz), 118.12 (d, *J* = 3.6 Hz), 113.40-112.70 (m), 110.55 (d, *J* = 25.6 Hz), 49.31, 39.50, 30.57, 30.30, 28.60, 16.92, 15.04

^{19}F NMR: -114.99 (1F, t, *J* = 9.9 Hz, ArF), -134.33 (2F, dd, *J* = 20.6 Hz, *J* = 8.8 Hz, ArF), -161.44 (1F, tt, *J* = 20.6 Hz, *J* = 6.6 Hz)

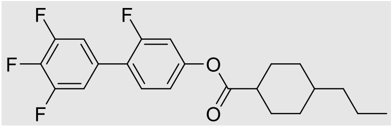

(**6**)

Yield: 350 mg (90 %, colourless crystals);

R_f (DCM):

^{1}H NMR (400 MHz): δ 7.27 (t, *J* = 8.7 Hz, 1H, Ar-**H**), 7.06 (ddd, *J* = 8.8, 6.5, 1.3 Hz, 2H, Ar-**H**), 6.91 – 6.84 (m, 2H, Ar-**H**), 2.40 (tt, *J* = 12.2, 3.6 Hz, 1H, Ar-C**H**-$(CH_2)_2$), 2.11 – 1.96 (m, 2H, CH-[(C$\mathbf{H_{eq}}$)H_{ax}]x2-CH_2), 1.85 – 1.73 (m, 2H, CH-[(C$\mathbf{H_{ax}}$)H_{eq}]x2-CH_2), 1.56 – 1.39 (m, 2H CH_2-[(C$\mathbf{H_{eq}}$)H_{ax}]x2-CH), 1.32 – 1.07 (m, 5H CH_2-[(C$\mathbf{H_{ax}}$)H_{eq}]x2-CH and CH_2CH-C$\mathbf{H_2}$-CH_2)*, 1.01 – 0.83 (m, 3H, CH_2-C**H**-CH_2 and CH_2-C$\mathbf{H_2}$-CH_3)*, 0.81 (t, *J* = 7.2 Hz, 3H, CH_2-C$\mathbf{H_3}$).

*overlapping signals

^{13}C{^{1}H} NMR (101 MHz, $CDCl_3$) [reported peaks]: 174.18, 160.55, 159.86, 151.76, 151.66, 130.41, 118.09, 113.03, 113.00, 110.64, 110.39, 77.31, 76.99, 76.67, 43.57, 39.38, 36.57, 32.14, 28.93, 19.88, 14.31.

^{19}F NMR (376 MHz): ^{19}F NMR (376 MHz, $CDCl_3$) δ -114.90 (t, *J* = 9.7 Hz, Ar-**F**), -134.31 (dd, *J* = 20.6, 8.8 Hz, Ar-**F**), -161.43 (tt, *J* = 20.6, 6.6 Hz, Ar-**F**).

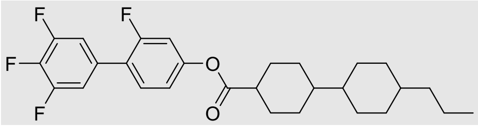

(**7**)

Yield: 285 mg (60 %, colourless crystals);

R_f (DCM): 0.84

^{1}H NMR (400 MHz): δ 7.36 (t, *J* = 8.7 Hz, 1H, Ar-**H**), 7.20 – 7.10 (m, 2H, Ar-**H**), 7.01 – 6.92 (m, 2H, Ar-**H**), 2.47 (tt, *J* = 12.2, 3.5 Hz, 1H, Ar-C**H**-(CH_2)$_2$), 2.21 – 2.09 (m, 2H, CH-[(C$\mathbf{H_{eq}}$)H_{ax}]x2-CH_2), 1.97 – 1.64 (m, 9H, CH-[(C$\mathbf{H_{ax}}$)H_{eq}]x2-CH_2, CH-[(C$\mathbf{H_{eq}}$)H_{ax}]x2-CH_2, and (CH_2)$_2$-CH-C**H**-(CH_2)$_2$)*, 1.64 – 1.46 (m, 2H), 1.39 – 1.24 (m, 3H), 1.23 – 0.93 (m, 11H), 0.93 – 0.76 (m, 5H).

^{13}C{^{1}H} NMR (101 MHz, $CDCl_3$) [reported peaks]: 174.20, 160.58, 158.09, 151.69, 130.42, 118.12, 118.09, 113.24, 113.05, 110.67, 110.41, 77.34, 77.02, 76.70, 43.65, 43.21, 42.48, 39.78, 37.59, 33.51, 30.01, 29.23, 29.08, 28.96, 28.76, 20.04, 14.42.

^{19}F NMR (376 MHz): δ -114.88 (t, *J* = 9.6 Hz. Ar-**F**), -134.28 (dd, *J* = 20.4, 8.9 Hz, Ar-**F**), -161.39 (tt, *J* = 20.8, 6.7 Hz, Ar-**F**).

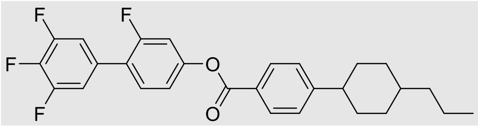

(**8**)

Yield: 300 mg (61 %, colourless crystals);

R_f (DCM): 0.80

^{1}H NMR (400 MHz): δ 8.12 (ddd, *J* = 8.4, 1.9, 1.8 Hz, 2H, Ar-**H**), 7.42 (t, *J* = 8.6 Hz, 1H, Ar-**H**), 7.37 (ddd, *J* = 8.2, 2.2, 1.8 Hz, 2H, Ar-**H**), 7.22 – 7.15 (m, 2H, Ar-**H**), 7.14 – 7.08 (m, 1H, Ar-**H**), 2.58 (tt, *J* = 12.0, 3.0 Hz, 1H, Ar-C**H**-(CH_2)$_2$), 1.91 (m, 5H, (CH_2)$_2$-CH-C$\mathbf{H_2}$-CH_2 and CH-[(C$\mathbf{H_{eq}}$)H_{ax}]x2-CH_2)*, 1.55 – 1.43 (m, 2H, CH-[(C$\mathbf{H_{ax}}$)H_{eq}]x2-CH_2), 1.43 – 1.28 (m, 2H, CH_2-[(C$\mathbf{H_{eq}}$)H_{ax}]x2-CH), 1.28 – 1.20 (m, 2H, CH_2-[(C$\mathbf{H_{ax}}$)H_{eq}]x2-CH), 1.16 – 1.00 (m, 2H, CH_2-C$\mathbf{H_2}$-CH_3), 0.92 (t, *J* = 7.2 Hz, 3H, CH_2-C$\mathbf{H_3}$).

*overlapping signals

^{13}C{^{1}H} NMR (101 MHz, $CDCl_3$) [reported peaks]: 164.73, 160.66, 158.16, 154.76, 152.42, 151.93, 151.82, 149.97, 140.74, 131.01, 130.57, 130.53, 130.44, 127.28, 126.39, 123.81, 123.68, 118.36, 118.32, 113.32, 113.28, 113.10, 110.91, 110.66, 44.90, 39.65, 36.97, 34.04, 33.38, 20.03, 14.40.

^{19}F NMR (376 MHz): δ -114.73 (t, *J* = 9.8 Hz, Ar-**F**), -134.25 (dd, *J* = 20.5, 8.7 Hz, Ar-**F**), -161.34 (tt, *J* = 20.8, 6.5 Hz, Ar-**F**).

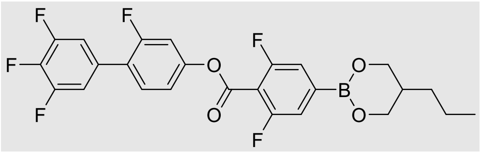

(**9 / BIO**)

Yield: 300mg (66%, colourless crystals);

R_f (DCM): 0.65

^{1}H NMR (400 MHz): 7.24 (2H, d, *J* = 9.3 Hz, ArH), 7.36-7.49 (3H, m, ArH), 4.10-4.30 (2H, m, B-[OCHH_{ax}CH-CHH_{ax}-O]), 7.14-7.23 (4H, m, ArH), 3.75-3.83 (2H, m, B-[OCHH_{eq}-CH-CHH_{eq}-O]), 2.00 – 2.02 (1H, m, B-[OCH$_2$-CH(-CH$_2$-CH$_2$...)-CH$_2$-O]), 1.35-1.46 (2H, m, CH-CH$_2$-CH$_2$-CH$_3$), 1.24-1.32 (2H, m, CH-CH$_2$-CH$_2$), 0.96 (3H, t, *J* = 7.2 Hz, z-CH$_2$-CH$_2$-C**H$_3$**)

^{13}C{^{1}H} NMR (101 MHz): 160.62, 160.35 (dd, *J* = 256.7 Hz, *J* = 26.1 Hz), 159.71, 158.12, 140.78 (t, *J* = 16.1 Hz), 138.27 (t, *J* = 15.0 Hz), 130.65 (d, *J* = 3.7 Hz), 124.25 (d, *J* = 12.8 Hz), 118.17 (d, *J* = 3.7 Hz), 116.81 (d, *J* = 3.7 Hz), 113.45-112.79 (m), 110.99 (d, *J* = 17.3 Hz), 110.66 (d, *J* = 26.1 Hz), 67.01 (BO-C-C), 36.15 (-OC**C**HCO-), 30.28 (HC-**C**H$_2$CH$_2$CH$_3$), 19.96 (CH$_2$**C**H$_2$CH$_3$), 14.15 (CH$_2$CH$_2$**C**H$_3$),

^{19}F NMR (376 MHz): -110.75 (2F, d, J_{F-H} = 9.5 Hz, ArF) , -114.44 (1F, t, J_{F-F} = 9.7 Hz, ArF), -134.26 (2F, J_{H-F} = 8.8 Hz, J_{F-F} = 20.7 Hz, ArF), -161.29 (2F, dd, J_{H-F} = 6.7 Hz, *J* = 20.7, ArF)

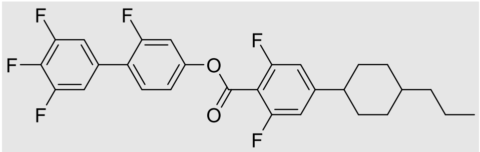

(**10 / CIO**)

Yield: 355 mg (71 %, colourless crystals);

R_f (DCM): 0.82

^{1}H NMR (400 MHz, CDCl3) δ 7.43 (t, J = 8.6 Hz, 1H, Ar-**H**), 7.22 – 7.10 (m, 5H, Ar-**H**), 6.89 (ddd, J = 10.7, 1.1, 1.1 Hz, 2H, Ar-**H**), 2.64 – 2.46 (m, 1H, Ar-C**H**-(CH$_2$)$_2$), 1.91 (ddd, J = 13.5, 6.4, 3.3 Hz, 5H, 2x (C**H$_{eq}$**)H_{ax}-CH-CH$_2$, (CH2)-C**H**-CH$_2$, and CH-C**H$_2$**-CH$_2$)*, 1.52 – 0.95 (m, 9H, 4x C**H$_2$**$_{cyclohexane}$ and CH2-CH$_2$-CH$_3$)*, 0.91 (t, J = 7.2 Hz, 3H, CH$_2$-C**H$_3$**).

*overlapping peaks

^{13}C{^{1}H} NMR (101 MHz, CDCl$_3$) [reported peaks]: δ 162.59, 162.53, 160.62, 160.02, 159.96, 159.58, 158.12, 156.27, 156.18, 151.15, 151.04, 130.64, 130.60, 124.12, 118.21, 118.17, 113.34, 113.31, 113.12, 113.09, 110.90, 110.87, 110.80, 110.68, 110.65, 110.54, 106.70, 44.64, 39.49, 36.81, 33.64, 33.07, 19.98, 14.36.

^{19}F NMR (376 MHz): δ -109.18 (d, *J* = 10.6 Hz, Ar-**F**), -114.45 (t, *J* = 9.7 Hz, Ar-**F**), -134.19 (dd, *J* = 20.6, 8.7 Hz, Ar-**F**), -161.20 (tt, *J* = 20.4, 6.6 Hz, Ar-**F**).

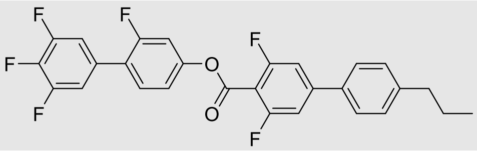

(**11 / PIO**)

Yield: 365 mg (73 %, colourless crystals);

R_f (DCM): 0.89

^{1}H NMR (400 MHz): δ 7.52 (ddd, *J* = 8.3, 2.0, 1.8 Hz, 2H, Ar-**H**), 7.44 (t, *J* = 8.6 Hz, 1H, Ar-**H**), 7.36 – 7.26 (m, 2H, Ar-**H**), 7.23 – 7.14 (m, 4H, Ar-**H**), 2.66 (t, *J* = 6.8 Hz, 2H, Ar-C**H₂**-CH₂), 1.69 (h, *J* = 7.5 Hz, 2H, CH₂-C**H₂**-CH₃), 0.98 (t, *J* = 7.3 Hz, 3H, CH₂-C**H₃**).

^{13}C{^{1}H} NMR (101 MHz, $CDCl_3$) [reported peaks]: δ 162.90, 160.64, 160.34, 160.27, 159.45, 158.14, 151.12, 151.01, 147.95, 144.58, 134.85, 130.69, 130.65, 129.41, 126.89, 118.21, 118.17, 113.36, 113.32, 113.14, 110.82, 110.60, 110.56, 110.37, 110.34, 37.72, 24.44, 13.81.

^{19}F NMR (376 MHz): δ -108.25 (d, *J* = 10.5 Hz, Ar-**F**), -114.36 (t, *J* = 9.8 Hz, Ar-**F**), -134.16 (dd, *J* = 20.7, 8.6 Hz, Ar-**F**), -161.16 (tt, *J* = 20.6, 6.6 Hz, Ar-**F**).

3.4 Example NMR Spectra

Fig. SX-SY are given as representative examples of the NMR spectra of **1-11**. The remaining NMR data is available at the attached DOI (https://doi.org/10.5518/1510).

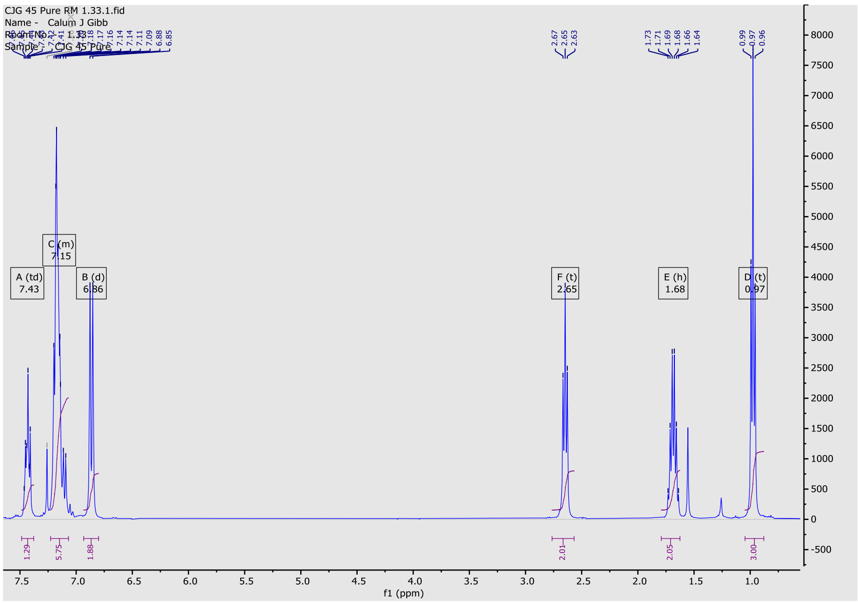

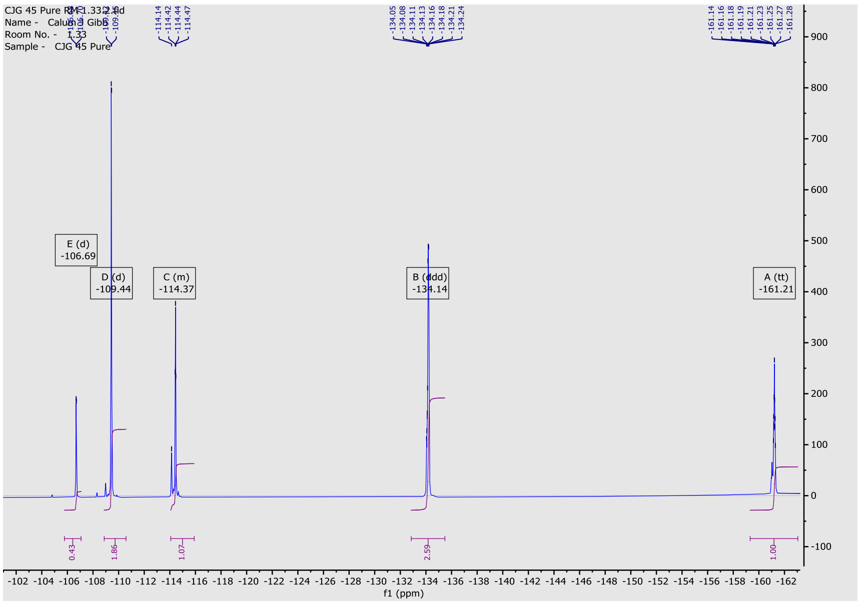

Fig. SX: E NMR spectra of **1**: ^{1}H [top], ^{19}F [middle], and $^{13}C\{^{1}H\}$ [bottom].

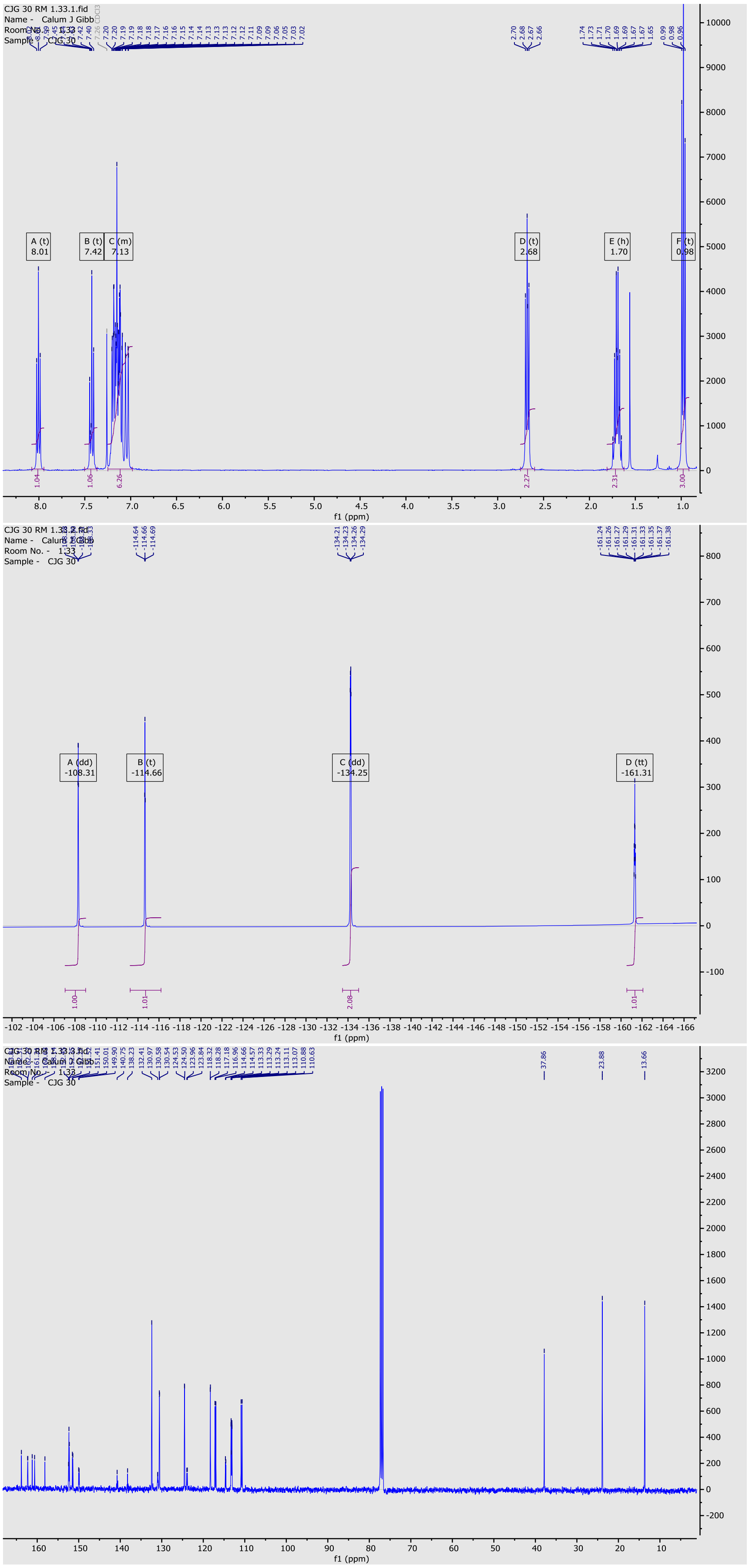

Fig. SX: E NMR spectra of **2**: ^{1}H [top], ^{19}F [middle], and $^{13}C\{^{1}H\}$ [bottom].

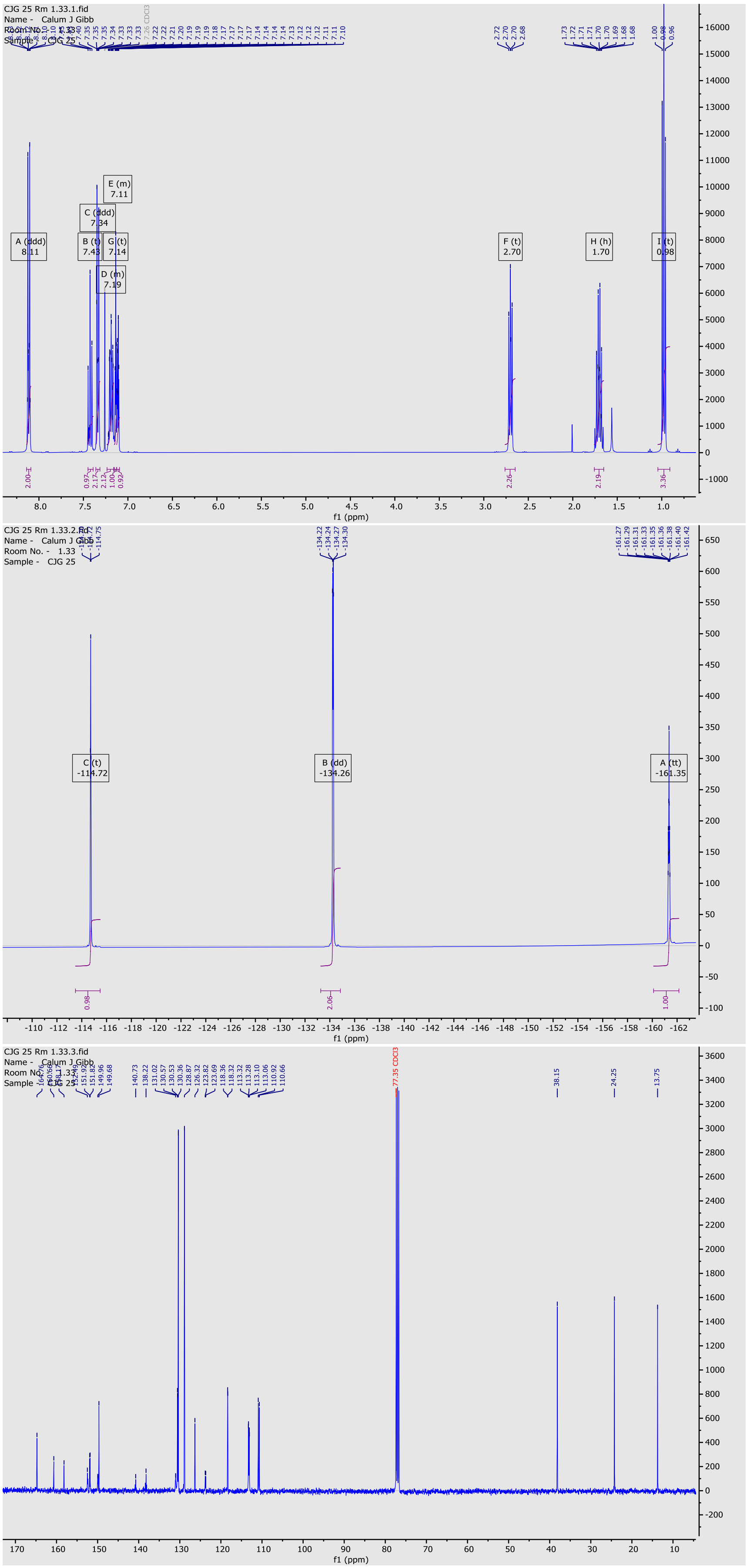

Fig. SX: E NMR spectra of **3**: ^{1}H [top], ^{19}F [middle], and ^{13}C{^{1}H} [bottom].

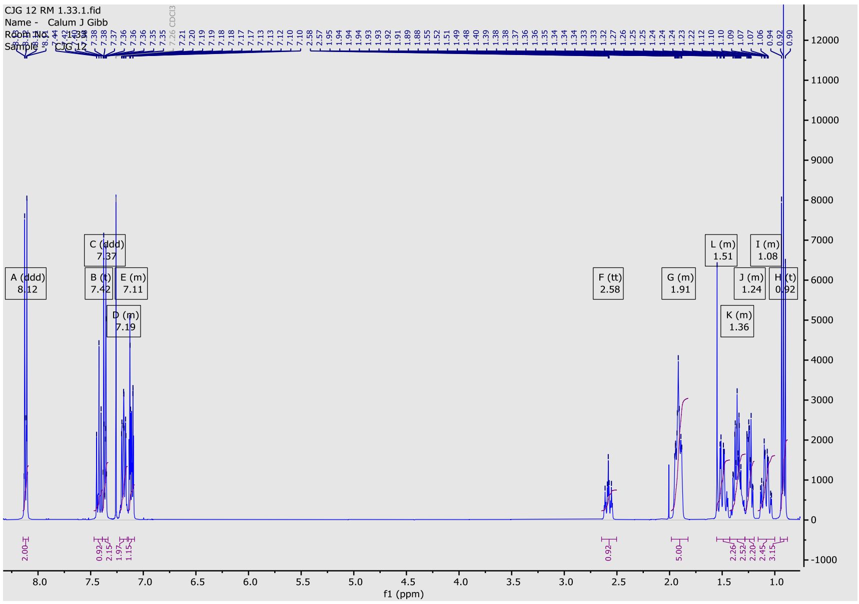
CJG 12 RM 1.33.1.fid
Name - Calum J Gibb
Room No. - 1.33
Sample - CJG 12
A (ddd) 8.12
B (t) 7.42
C (ddd) 7.37
D (m) 7.19
E (m) 7.11
F (tt) 2.58
G (m) 1.91
H (t) 0.92
I (m) 1.08
J (m) 1.24
K (m) 1.36
L (m) 1.51
f1 (ppm)

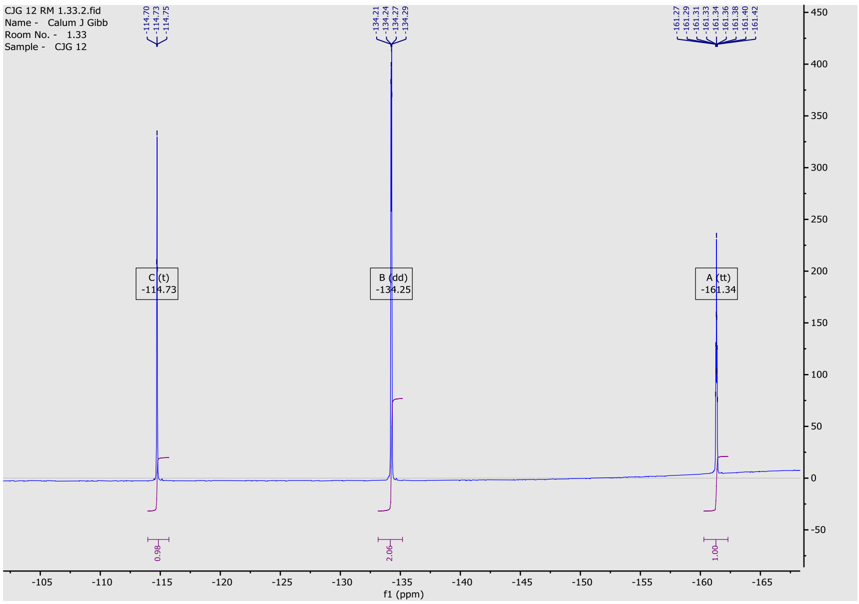
CJG 12 RM 1.33.2.fid
Name - Calum J Gibb
Room No. - 1.33
Sample - CJG 12
C (t) -114.73
B (dd) -134.25
A (tt) -161.34
f1 (ppm)

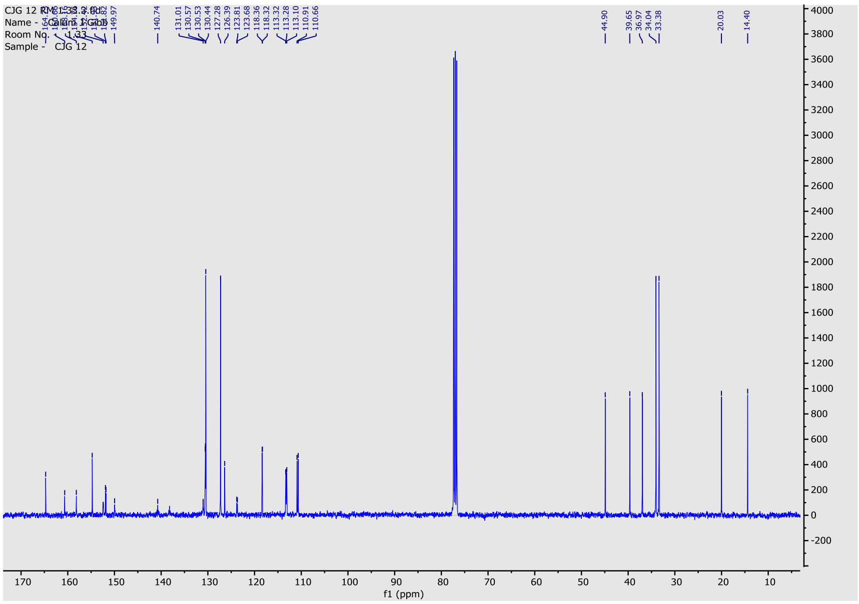
Name - Calum J Gibb
Room No. - 1.33
Sample - CJG 12
140.74
131.01
130.57
130.53
130.44
127.28
126.39
123.81
123.68
118.36
118.32
113.32
113.28
113.10
110.91
110.66
44.90
39.65
36.97
34.04
33.38
20.03
14.40
f1 (ppm)

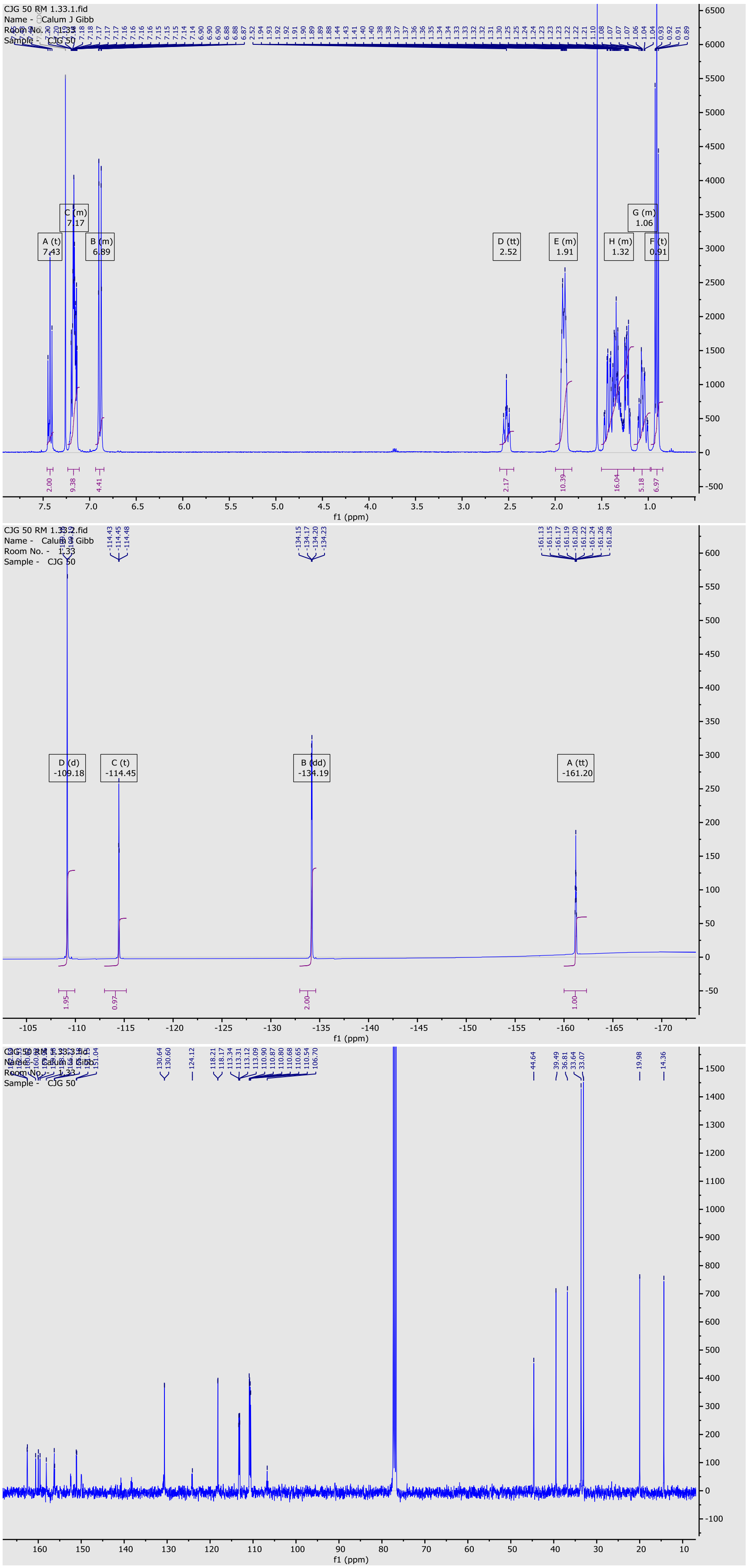

Fig. SX: E NMR spectra of **10 / ClO**: ^{1}H [top], ^{19}F [middle], and ^{13}C{^{1}H} [bottom].

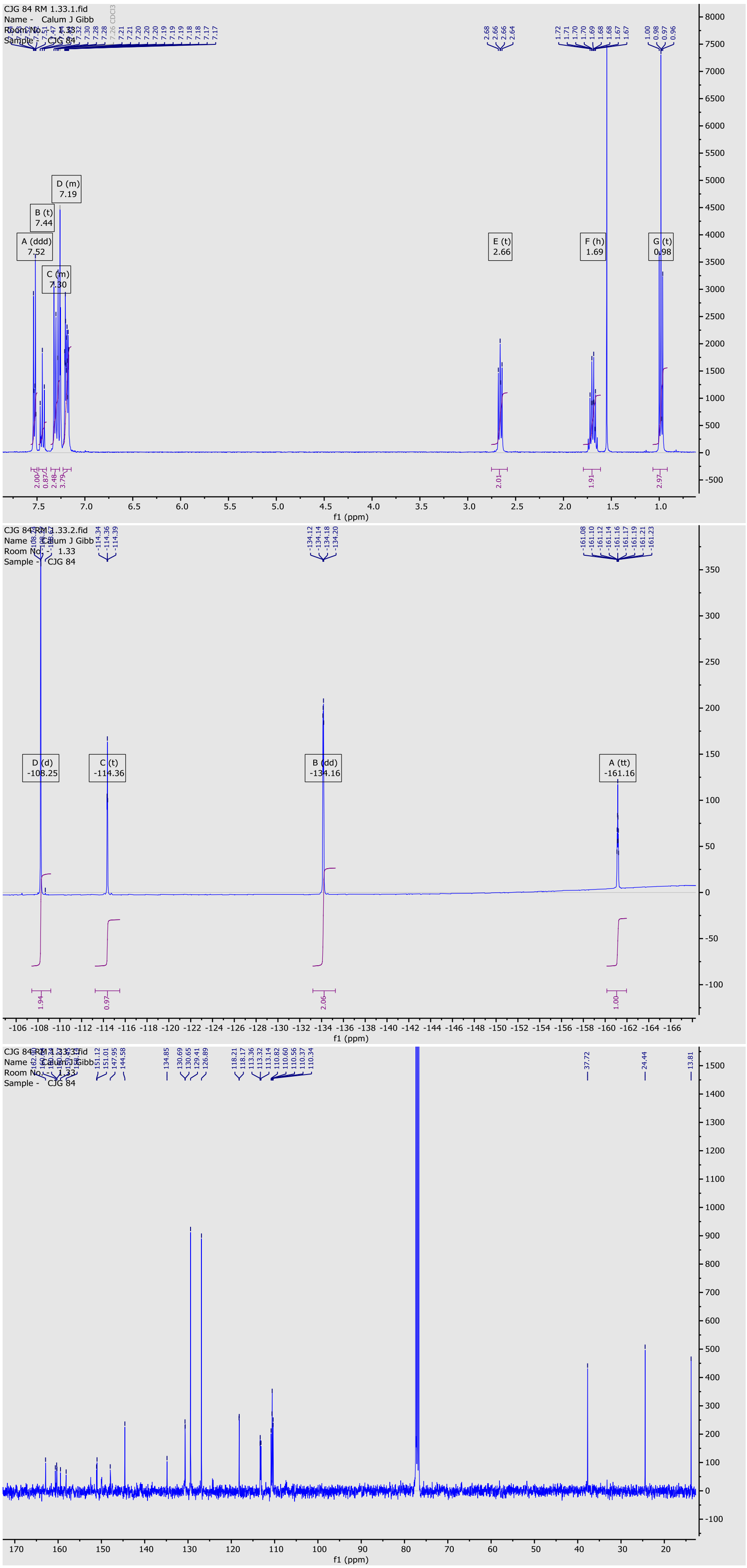

Fig. SX: E NMR spectra of **11 / PIO**: ^{1}H [top], ^{19}F [middle], and ^{13}C{^{1}H} [bottom].

4 Supplemental References

[1] M. J. Frisch, G. W. Trucks, H. B. Schlegel, G. E. Scuseria, M. A. Robb, J. R. Cheeseman, G. Scalmani, V. Barone, B. Mennucci, G. A. Petersson, H. Nakatsuji, M. Caricato, X. Li, H. P. Hratchian, A. F. Izmaylov, J. Bloino, G. Zheng, J. L. Sonnenberg, M. Hada, M. Ehara, K. Toyota, R. Fukuda, J. Hasegawa, M. Ishida, T. Nakajima, Y. Honda, O. Kitao, H. Nakai, T. Vreven, J. A. Montgomery Jr., J. E. Peralta, F. Ogliaro, M. J. Bearpark, J. Heyd, E. N. Brothers, K. N. Kudin, V. N. Staroverov, R. Kobayashi, J. Normand, K. Raghavachari, A. P. Rendell, J. C. Burant, S. S. Iyengar, J. Tomasi, M. Cossi, N. Rega, N. J. Millam, M. Klene, J. E. Knox, J. B. Cross, V. Bakken, C. Adamo, J. Jaramillo, R. Gomperts, R. E. Stratmann, O. Yazyev, A. J. Austin,R. Cammi, C. Pomelli, J. W. Ochterski, R. L. Martin, K. Morokuma, V. G. Zakrzewski, G. A. Voth, P. Salvador, J. J. Dannenberg, S. Dapprich, A. D. Daniels, O. Farkas, J. B. Foresman, J. V. Ortiz, J. Cioslowskiand, D. J. Fox, Gaussian 016, Revision E.01, Gaussian, Inc., Wallingford CT, 2016.

[2] T. H. Dunning, Gaussian Basis Sets for Use in Correlated Molecular Calculations. I. The Atoms Boron through Neon and Hydrogen, J Chem Phys 90, 1007 (1989).

[3] S. Grimme, S. Ehrlich, and L. Goerigk, Effect of the Damping Function in Dispersion Corrected Density Functional Theory, J Comput Chem 32, 1456 (2011).

[4] A. D. Becke, Density-Functional Thermochemistry. III. The Role of Exact Exchange, J Chem Phys 98, 5648 (1993).

[5] S. Wang, J. Witek, G. A. Landrum, and S. Riniker, Improving Conformer Generation for Small Rings and Macrocycles Based on Distance Geometry and Experimental Torsional-Angle Preferences, J Chem Inf Model 60, 2044 (2020).

[6] Martinot-Lagarde Ph., Direct Electrical Measurement of the Permanent Polarization of a Ferroelectric Chiral Smectic C Liquid Crystal, J. Physique Lett. 38, 17 (1977).

[7] K. Miyasato, S. Abe, H. Takezoe, A. Fukuda, and E. Kuze, Direct Method with Triangular Waves for Measuring Spontaneous Polarization in Ferroelectric Liquid Crystals, Jpn J Appl Phys 22, L661 (1983).

[8] X. Chen et al., The Smectic ZA Phase: Antiferroelectric Smectic Order as a Prelude to the Ferroelectric Nematic, PNAS 120, (2023).
